# PHLPP1 Regulates Inflammatory Signaling in Degenerated Nucleus Pulposus Cells in Mice and Humans

**DOI:** 10.3390/cells15151362

**Published:** 2026-07-29

**Authors:** Biplab Chatterjee, Nazir M. Khan, Tushar Singh, Neil Romesh, Seong Hee Her, Changli Zhang, Hicham Drissi, Svenja Illien-Jünger

**Affiliations:** 1 Department of Orthopaedics, Center for Musculoskeletal Research, Emory University School of Medicine, Atlanta, GA 30329, USA; biplab.chatterjee@emory.edu (B.C.); mnkhan2@emory.edu (N.M.K.); tsing47@emory.edu (T.S.); neilromesh@gmail.com (N.R.); seong.hee.her@emory.edu (S.H.H.); changli.zhang@emory.edu (C.Z.); hicham.drissi@emory.edu (H.D.); 2 Atlanta Veterans Affairs Medical Center, Decatur, GA 30033, USA; 3 Wallace H. Coulter Department of Biomedical Engineering, Georgia Institute of Technology, Atlanta, GA 30332, USA

**Keywords:** intervertebral disc, IVD, nucleus pulposus, NP, PHLPP1, mouse model of spontaneous IVD degeneration, inflammation, cytokines, chemokines, human NP cell culture, RNA sequencing, STAT1

## Abstract

**Highlights:**

**What are the main findings?**

- Phlpp1 deficiency attenuated age-related intervertebral disc degeneration and inflammation in mice, which were most noticeable in males.
- *PHLPP1* knockdown in degenerated human nucleus pulposus cells significantly suppresses inflammatory and chemokine gene expression (e.g., *IL1B*, *IL6*, *CXCL2*, *CCL20*, *STAT1*) while promoting ECM-associated gene expression, indicating a shift toward a less catabolic phenotype.

**What are the implications of the main findings?**

- *PHLPP1* functions as a regulator of pro-inflammatory and catabolic signaling in nucleus pulposus cells, suggesting that it contributes to the progression of intervertebral disc degeneration.
- Targeting *PHLPP1* may represent a potential therapeutic strategy to reduce inflammation and preserve matrix homeostasis in degenerative disc degeneration.

**Abstract:**

Intervertebral disc degeneration is a major contributor to discogenic low back pain and is characterized by a pro-inflammatory and catabolic microenvironment within the nucleus pulposus. We previously showed that the phosphatase PH domain leucine-rich repeat protein phosphatase 1 (PHLPP1) is positively correlated with intervertebral disc degeneration and that its deficiency promoted nucleus pulposus cell survival and matrix homeostasis; however, its role in inflammatory signaling during intervertebral disc degeneration remained unknown. Here, we show that PHLPP1 functions as an upstream regulator of inflammatory and catabolic networks in the degenerating intervertebral disc. In aged mice, *Phlpp1* deficiency attenuated spontaneous intervertebral disc degeneration and reduced expression of IL1B and IL6. Notably, severe age-associated degeneration and inflammation were observed primarily in male wildtype mice, where the protective effects of *Phlpp1* deletion were most apparent. To define the underlying mechanisms in human disease, we performed transcriptomic profiling of degenerated human nucleus pulposus cells following siRNA-mediated *PHLPP1* silencing. *PHLPP1* depletion induced widespread transcriptional reprogramming characterized by suppression of inflammatory cytokines, chemokines, and matrix-degrading enzymes, while promoting expression of extracellular matrix-associated genes. Pathway enrichment and network analyses identified coordinated inhibition of cytokine–cytokine receptor interaction, TNF, IL17, chemokine, and NFKB signaling pathways, revealing PHLPP1 as a central node linking cytokine amplification, immune cell recruitment, and matrix degradation. These findings were validated by reduced expression of *IL1A*, *IL1B*, *IL6*, *CXCL2*, *CCL20*, and *STAT1*, alongside increased *ACAN* expression. Furthermore, *PHLPP1* silencing attenuated IL1B-induced inflammatory activation and matrix suppression and reduced STAT1 phosphorylation after IL1B stimulation. Collectively, our findings identify PHLPP1 as a critical regulator coupling inflammatory amplification to matrix remodeling in degenerating intervertebral discs and nominate PHLPP1 inhibition as a potential disease-modifying therapeutic strategy for intervertebral disc degeneration.

## 1. Introduction

Low back pain is a highly prevalent clinical condition, affecting more than 600 million people globally [1]. Discogenic low back pain accounts for 26–42% of all back pain cases [2] and represents the most common underlying diagnosis among individuals with low back pain [3,4]. Well-known risk factors for intervertebral disc (IVD) degeneration (IDD) include aging, genetic predisposition, aberrant mechanical loading, a sedentary lifestyle, and poor diet [5,6]. IVDs consist of three distinct tissues: the nucleus pulposus (NP), a hydrated, gel-like core enriched with the proteoglycan Aggrecan, which binds water and enables the tissue to withstand compressive forces; the fibrocartilaginous annulus fibrosus, which consists of concentric lamellar layers that provide mechanical stability [7,8]; and the cartilaginous endplates that anchor the IVDs to adjacent vertebral bodies [9]. Signs of spontaneous IDD first occur in the NP and are characterized by increased production of pro-inflammatory cytokines and progressive extracellular matrix (ECM) degradation by increasing the expression of matrix metalloproteases (MMPs). Major cytokines implicated in this process include tumor necrosis factor-α (TNFA), interleukins, and chemokines [10,11,12]. Among cytokines, TNFA, IL1B, and IL6 are the most extensively characterized in IVDs [13,14,15]. This pro-inflammatory and catabolic environment further accelerates IDD by increasing the expression of ECM-degrading enzymes [14,16,17] and phosphatases [18,19,20].

We previously showed that the phosphatase PH domain leucine-rich repeat protein phosphatase 1 (PHLPP1) is highly expressed in degenerated human IVD tissues [21] and that its deficiency in mice decelerated age- and injury-induced IDD by promoting ECM production and cell survival, while decreasing apoptotic cell death [21,22]. In other musculoskeletal tissues, PHLPP1 has been shown to induce osteoclast-mediated bone resorption [23], cartilage degradation [24], and post-traumatic osteoarthritis [25]. Consistent with these catabolic effects, evidence also supports an association between PHLPP1 and inflammation in osteoarthritis [25] and bone homeostasis [26]. Building on these observations, the aim of the present study was to determine whether PHLPP1 functions as an upstream regulator of inflammatory signaling networks in NP cells, and if its deficiency can inhibit inflammatory signaling and catabolic pathways in NP cells. We examined lumbar IVDs from aged *Phlpp1* global knockout (KO) and wild-type (WT) mice for age-related IDD. Bulk RNA sequencing was performed to assess the transcriptomic profile of degenerated human NP cells after *PHLPP1* knockdown, and the results were validated using RT-qPCR. Degenerated human NP cell cultures were treated with IL1B to simulate the pro-inflammatory environment of degenerated IVDs and to assess the protective effect of *PHLPP1* knockdown against inflammatory stimuli.

## 2. Materials and Methods

### 2.1. Study Design

**Figure 1:**
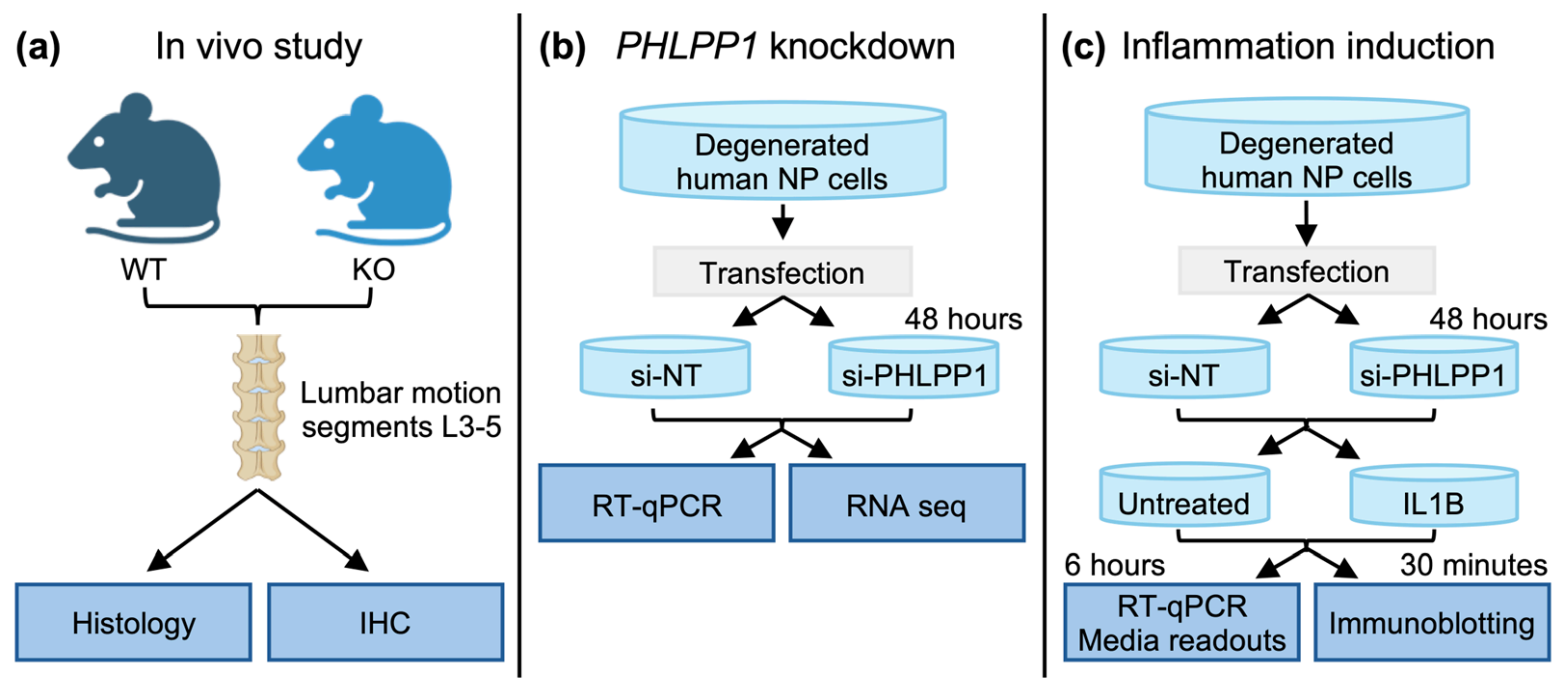
Study Design. (**a**) Lumbar IVDs from 20-month-old female and male WT and KO mice were collected for histological and immunohistochemical analyses. (**b**) Degenerated human NP cells from female and male donors were cultured to ~90% confluency and transfected with non-targeting siRNA (si-NT) or *PHLPP1* siRNA (si-PHLPP1). After 72 h, RNA was isolated for bulk RNA-seq and RT-qPCR analyses. (**c**) Degenerated human NP cells were cultured to ~90% confluency and serum-starved (0.2% DMEM, 2 h), followed by transfection with si-NT or si-PHLPP1. After 48 h, cells were treated with IL-1β (10 ng/mL), and RNA, culture media (6 h) and protein (30 min) were collected for RT-qPCR analysis.

Degenerated human NP cells and IVDs of aged *Phlpp1* knockout mice were used to determine the role of PHLPP1 as an upstream regulator of inflammatory signaling networks during IDD (Figure 1). Mouse lumbar spines were harvested from 20-month-old female and male WT and *Phlpp1* KO mice and processed for IDD and immunohistochemical analysis of IL1B and IL6. Degenerated human NP cells were transfected with either non-targeting small interfering RNA (si-NT) or *PHLPP1*-targeting siRNA (si-PHLPP1) to assess the transcriptomic profile after *PHLPP1* knockdown and to identify whether *PHLPP1* deficiency inhibits inflammatory signaling after IL1B stimulation.

### 2.2. Mouse Model of Spontaneous IDD

All animal experiment procedures complied with the National Institutes of Health (NIH) Guide for the Care and Use of Laboratory Animals (U.S. Department of Health, Education, and Welfare, NIH 78-23, 1996) and were carried out under protocols approved by the Institutional Animal Care and Use Committee (IACUC) of the Atlanta Veterans Affairs Medical Center (Protocol V020-22). This study utilized mouse cohorts from a previous study [22]. Mice were housed with 12 h of light/dark cycles and given food and water ad libitum. Mouse igloos were used as environmental enrichment. C57BL/6J WT and *Phlpp1* global KO mice (WT: n = 5/6 females/males; KO: n = 8/5 females/males) were sacrificed at 20 months, when signs of spontaneous IDD have become morphologically visible [27]. Mice were excluded from the final data analysis if they died prematurely or required euthanasia before the completion of the study. Consequently, n = 2 female WT mice, n = 3 male WT mice and n = 2 male KO mice were excluded from downstream analysis due to early mortality. Following euthanasia, lumbar IVDs (L3-L6) were harvested and processed for histological and immunohistochemical evaluation (1–3 IVDs/mouse; Table 1). The number of analyzed IVDs varied due to the limited availability of the remaining histological sections from the previous study.

### 2.3. Histology and Immunohistochemistry (IHC)

IVDs were fixed in z-fix (Sigma Aldrich, St. Louis, MO, USA, Z2902-3.75L) for 2 days and decalcified in 5% formic acid (Millipore Sigma, Burlington, MA, USA, FX0440-6) for 3 days. Decalcified IVDs were embedded in paraffin, and 5 μm thick mid-coronal sections were used for histology (picrosirius red/alcian blue (PR/AB)) and IHC for IL1B and IL6. For each staining, all sections were processed at the same time. For IHC, antigen retrieval was performed by applying 0.8% hyaluronidase (Sigma Aldrich, H3506) for 1 h at 37 °C, followed by 30 min incubation in blocking buffer (2.5% normal horse serum; ImmPress–AP Horse Anti-Rabbit IgG Polymer Reagent, MP- 5401-50, Vector Laboratories, Newark, CA, USA). Samples were then separately incubated with primary antibodies against IL1B (Novus, Centennial, CO, USA, NB600-633) and IL6 (Novus, NB600-1131). After overnight incubation at 4 °C, the samples were washed and incubated with an alkaline phosphatase-conjugated secondary antibody (ImmPress–AP Horse Anti-Rabbit IgG Polymer Reagent, Vector Laboratories, MP-5401-50) for 30 min and visualized using alkaline phosphatase substrate (ImmPACT Vector Red, Vector Laboratories, SK-5105). All samples were counterstained with hematoxylin (Electron Microscopy Sciences, Hatfield, PA, USA, 26041-05) and mounted with Permount (Fisher Chemical, Pittsburgh, PA, USA, SP15-500). Universal negative control serum (NC498, BioCare Medical, Pacheco, CA, USA) was used as a negative control. Sections were imaged by bright-field microscopy (DM6 B automated microscope, LAS X software 3.6.0.20104; Leica, Germany) using consistent settings for each stain. IL-1β- and IL-6–positive areas were quantified in ImageJ (FIJI; [28], National Institutes of Health, Bethesda, MD, USA) by outlining the NP region of interest (ROI) and applying Color Deconvolution to separate hematoxylin and ImmPACT Vector Red signals into grayscale channels. The Fast Red channel was used to calculate the stained area within the NP. Thresholds were defined using negative controls and sections with minimal and maximal staining and were uniformly applied across all images to ensure consistent quantification within each dataset. One section per IVD was obtained for each stain, and IDD grade/immunopositively areas were quantified by two independent observers who were blinded to experimental groups. IVDs were graded using the Tam histopathological grading system [29].

### 2.4. Human NP Cell Extraction and Culture

After informed patient consent and following a protocol approved by the Institutional Review Board at the Emory University School of Medicine (IRB #00099028, approved 18 May 2021), degenerated human NP tissues from female and male donors (Grade 3–4 on the Pfirrman grade [30]; Table 2) were collected after discectomy surgeries for discogenic back pain. Only NP tissues that could clearly be distinguished from annulus fibrosus tissues and did not contain herniated or unidentifiable tissues were used for NP cell isolation as previously described [31]. Briefly, NP tissues were cut into 1–2 mm^3^ pieces, rinsed in phosphate-buffered saline, and enzymatically released using 0.2% protease (Sigma Aldrich, ST. Louis, MO, USA, P5147-1G) for 1 h followed by 0.025% collagenase P (Roche Diagnostics, Mannheim, Germany, 40341623) for 4 h. Isolated NP cells were expanded and maintained in low glucose DMEM (Gibco, 11885084) containing 10% FBS, 1% pen/strep, and 50 µg/mL L-Ascorbic acid 2-phosphate (Sigma, A8960) and cultured at 37 °C under 5% CO_2_, normoxia, and 90% humidity. Passage 3 cells were used for all experiments. Low-glucose DMEM was used for all experiments.

### 2.5. Cell Transfection

Cell transfection of degenerated human NP cells was performed as previously described [22]. Briefly, NP cells were plated in 6-well culture plates and cultured until reaching 90% confluency. Knockdown of *PHLPP1* was performed using 25 nM ON-TARGETplus *PHLPP1* siRNA SMARTpool (L-019103-00-0005, Horizon, Cambridge, UK), with a non-targeting siRNA (D-001810-01-05, Horizon, Cambridge, UK) serving as control. For transfection, siRNA and DharmaFECT1 transfection reagent (T-2002-01, Horizon, Cambridge, UK) were each diluted in serum-free medium, incubated separately for 5 min at room temperature, then combined and incubated for additional 20 min to allow complex formation. Antibiotic-free low-serum medium (1% FBS) was added to bring the final volume to 2 mL, and the transfection mixture was applied to the cells for 24 h in medium. To minimize the risk of cytotoxicity from transfection, the medium was replaced with fresh DMEM supplemented with 10% FBS. Cells were then maintained for an additional 48 h before harvesting RNA.

### 2.6. IL1B Stimulation After Cell Transfection

Degenerated human NP cells were expanded into 12-well culture plates until reaching 90% confluency. Prior to transfection, cells were incubated under low-serum conditions (0.2% serum) for 2 h. Knockdown of *PHLPP1* was achieved as described above. 48 h after transfection, cells were stimulated with recombinant human *IL1B* (10 ng/mL, under serum-deprived conditions; 201-LB, R&D Systems, Minneapolis, MN, USA) for 30 min (immunoblotting) or 6 h (RNA extraction and Culture media).

### 2.7. RNA Isolation

Total RNA was extracted using TRIzol Reagent (Thermo Fisher Scientific, Waltham, MA, USA, 15596026) in combination with chloroform (Thermo Fisher Scientific, AAJ67241AP). The aqueous phase was subsequently purified using the RNeasy Micro Kit (Qiagen, Germantown, MD, USA, 74004) following the manufacturer’s instructions. RNA yield and purity were evaluated using the Qubit 4 Fluorometer with the Qubit dsDNA HS Assay Kit (Invitrogen, Carlsbad, CA, USA, Q32852) as per the manufacturer’s instructions. RNA integrity was further evaluated using the Agilent 2200 Bioanalyzer (Santa Clara, CA, USA) and only samples with RNA integrity number (RIN) ≥ 7.0 were used for downstream sequencing analysis [32,33,34]. After RIN analysis, three biological samples per treatment group derived from four independent human donors were used for library preparation (Table 2).

### 2.8. Library Preparation and Bulk RNA Sequencing

RNA sequencing libraries were generated from 250 ng of total RNA using the TruSeq Stranded Total RNA Library Preparation Kit (Illumina, San Diego, CA, USA) following the manufacturer’s protocol [35]. Polyadenylated transcripts were enriched using the poly(A) selection method, and directional libraries were generated using the NEBNext Ultra II RNA library preparation workflow. The resulting libraries were quantified, pooled, and sequenced using an Illumina sequencing platform, generating paired-end reads (2 × 150 bp) with an average depth of approximately 40 million reads per sample. Sequencing was performed at the Admera Health Sequencing Facility (South Plainfield, NJ, USA).

### 2.9. RNA-Seq Data Preprocessing and Quality Control

Raw sequencing reads were subjected to quality control analysis to evaluate base quality scores, GC content distribution, and sequencing error rates. Adapter sequences and low-quality reads were removed using standard preprocessing pipelines to generate high-quality clean reads. Clean reads were then aligned to the human reference genome (GRCh38) using a splice-aware alignment algorithm. Gene expression levels were quantified and normalized as Fragments Per Kilobase of transcript per Million mapped reads (FPKM) to account for transcript length and sequencing depth [36].

### 2.10. Differential Gene Expression Analysis

Differential gene expression analysis was performed using the DESeq2 package within the R/Bioconductor framework [37]. Gene counts were normalized across samples using the DESeq2 normalization algorithm to account for library size and sequencing depth. Differentially expressed genes (DEGs) between si-PHLPP1 and si-NT groups were identified based on the criteria using fold change ≥1.5 and adjusted *p*-value (False Discovery Rate, FDR) < 0.05 [37]. Multiple testing correction was applied using the Benjamini–Hochberg method to control the false discovery rate [33]. Fold change values were calculated from normalized gene expression counts to determine the magnitude of transcriptional changes following *PHLPP1* knockdown. Differential gene expression analysis was performed using the DESeq2 package within the R/Bioconductor framework [37]. Gene counts were normalized across samples using the DESeq2 normalization algorithm to account for library size and sequencing depth. Differentially expressed genes (DEGs) between siPHLPP1 and siNT groups were identified based on the criteria of fold change ≥ 1.5 and adjusted *p*-value (False Discovery Rate, FDR) < 0.05 [37]. Multiple testing correction was applied using the Benjamini–Hochberg method to control the false discovery rate [33]. Fold change values were calculated from normalized gene expression counts to determine the magnitude of transcriptional changes following *PHLPP1* knockdown.

### 2.11. Principal Component Analysis (PCA) and Hierarchical Clustering

PCA was performed using the R/Bioconductor statistical environment to assess global transcriptomic differences between si-PHLPP1 and si-NT samples. During preprocessing, gene expression matrices were subjected to unit variance scaling, and principal components were computed using singular value decomposition (SVD). PCA plots were generated to visualize clustering patterns and variability among biological replicates [38]. Hierarchical clustering and heatmap visualization were performed using normalized expression values. Rows were centered and scaled to unit variance, and clustering was performed using correlation distance with average linkage method to identify global gene expression patterns across samples.

### 2.12. Gene Ontology (GO) Enrichment Analysis

GO enrichment analysis was performed to identify significantly enriched biological processes (BP) and molecular functions (MF) among differentially expressed genes (DEGs). Enrichment analysis was conducted using the STRING (Search Tool for the Retrieval of Interacting Genes/Proteins) database [39]. The statistical significance of enriched GO terms was determined using the cumulative hypergeometric test. Multiple testing correction was performed using FDR adjustments. GO terms with FDR < 0.05 were considered significantly enriched.

### 2.13. Kyoto Encyclopedia of Genes and Genomes (KEGG) Pathway Enrichment Analysis

To identify biological signaling pathways associated with *PHLPP1*-regulated genes, KEGG pathway enrichment analysis was performed using the STRING database and R-based enrichment tools. Enrichment significance was determined using the hypergeometric distribution test, and pathways with FDR < 0.05 were considered statistically significant.

### 2.14. Protein–Protein Interaction (PPI) Network Analysis

To investigate the functional relationships among DEGs, PPI network analysis was performed using the STRING database. Genes significantly downregulated following *PHLPP1* knockdown were mapped to known protein interaction networks [40]. The resulting interaction network allowed identification of highly interconnected gene clusters and functional modules involved in inflammatory signaling, chemokine activity, and matrix remodeling.

### 2.15. RT-qPCR

Quantitative PCR (qPCR) reactions were performed in a total volume of 20 μL containing 8 ng cDNA, gene-specific primers (Table 3), 2× PowerUp SYBR Green Master Mix (Thermo Fisher Scientific, A25742), and RNase-free water. Amplification and detection were conducted using the CFX Opus 96 Real-Time PCR System (Bio-Rad). Beta-actin was used as the endogenous reference control. Relative gene expression levels were calculated using the 2^−^ΔΔCt^ method [41].

### 2.16. Quantification of IL6 in Conditioned Media by ELISA

The concentration of IL6 in the conditioned medium was quantified using the Quantikine^®^ Human IL6 ELISA Kit (R&D systems, Minneapolis, MN, USA, D6050) following the manufacturer’s instructions. Briefly, 100 μL of assay diluent and 100 μL of standards, controls, or samples were added to each well and incubated for 2 h at room temperature. Following washing, 200 μL of Human IL6 Conjugate was added and incubated for an additional 2 h. After the second wash, 200 μL of substrate solution was added to each well and incubated for 20 min at room temperature in the dark. The reaction was terminated by adding 50 μL of stop solution. Absorbance was measured at 450 nm with wavelength correction at 540 nm using a Promega GloMax^®^ Discover microplate reader (Promega Corporation, Madison, WI, USA, Serial No. 9700100288).

### 2.17. Measurement of Nitrite Content in Conditioned Media by Griess Assay

Nitrite levels were measured using the Griess assay which serves as an indirect indicator of inflammatory activity, as nitric oxide produced by inducible nitric oxide synthase during inflammation and is rapidly converted to stable nitrite in culture media. The assay was performed according to the manufacturer’s instructions (Catalog No. G7921). Briefly, 130 μL of deionized water, 20 μL of Griess reagent, and 150 μL of nitrite-containing samples or standards were added to each well of a microplate and incubated for 30 min at room temperature. A reference blank containing 20 μL of Griess reagent and 280 μL of deionized water was prepared in parallel. Absorbance was measured relative to the reference blank within the 520–590 nm range, using a Promega GloMax^®^ Discover microplate reader (Promega Corporation, Madison, WI, USA, Serial No. 9700100288).

### 2.18. Immunoblotting

Equal amounts of total protein (10 µg) were separated by SDS–PAGE and transferred onto PVDF membranes. Membranes were blocked using 5% (*w*/*v*) non-fat dry milk (Bio-Rad, catalog no- 1706404) in TBST (Tris-Buffered Saline + 0.1% Tween-20) and incubated with primary antibodies against PHLPP1 (Sigma, St. Louis, MO, USA, 071341), STAT1 (Cell Signaling Technology, Danvers, MA, USA, 14994T), Phospho-STAT1 (Ser-727; Cell Signaling Technology, Danvers, MA, USA, 8826T), and beta-actin (ACTB; Cell Signaling Technology, 5125S), followed by HRP-conjugated secondary antibodies (Fisher Scientific, PI32460). Immunoreactive bands were detected using ECL Western blotting substrate (Life Technologies, Carlsbad, CA, USA, 32106) and quantified by densitometric analysis using ImageJ 2.16.0/1.54p as previously described [22].

### 2.19. Statistics

Statistical analysis of RNA-seq data was performed using the DESeq2 package in R/Bioconductor. Statistical significance was assessed using a negative binomial generalized linear model, and *p*-values were adjusted for multiple comparisons using the Benjamini–Hochberg FDR method. Genes with an adjusted *p*-value (FDR) < 0.05 and fold change ≥ 1.5 were considered significantly differentially expressed. For gene ontology (GO) and KEGG pathway enrichment analyses, statistical significance was determined using the hypergeometric test, with FDR correction applied for multiple testing. Pathways with FDR < 0.05 were considered significantly enriched. For RT-qPCR, paired one-way analysis of variance (ANOVA) was used for comparisons between groups. Mouse IHC data were analyzed using two-way ANOVA to assess the effect of genotype and sex on IDD. Significant differences between groups were assessed by Šidák post hoc testing. Statistical analyses were performed using GraphPad Prism 11 (GraphPad Software, Inc., La Jolla, CA, USA). RT-qPCR and IHC data were presented as median with interleaved scatter blots. Statistical significance was defined as *p*-value < 0.05.

## 3. Results

### 3.1. Male Phlpp1 Knockout Mice Exhibited Reduced Age-Related Spontaneous IDD

**Figure 2:**
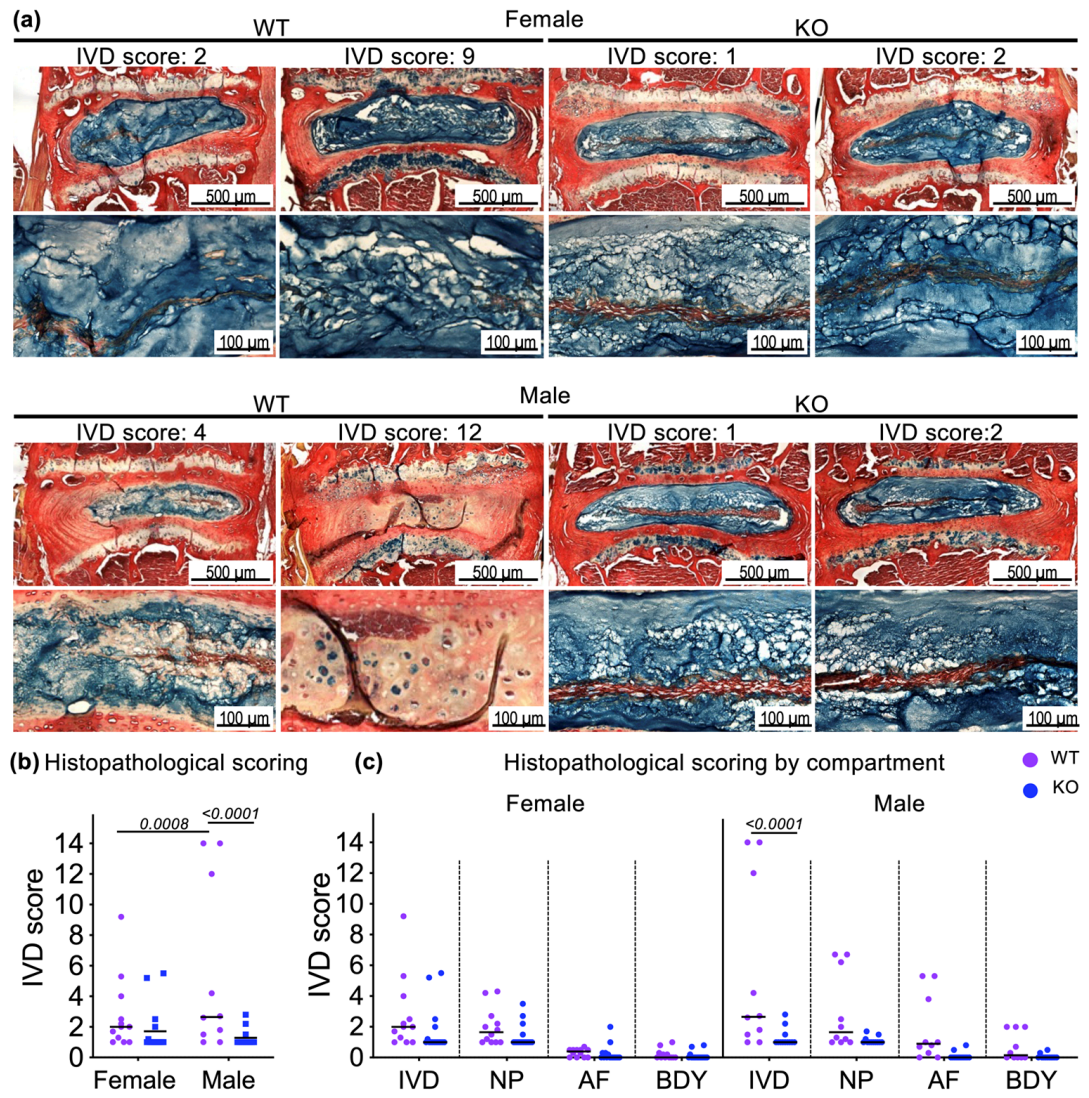
*Phlpp1* deficiency attenuated spontaneous, age-associated IDD in male mice. (**a**) Picrosirius red/alcian blue (PR/AB) staining was performed on IVDs from wild-type (WT) and knockout (KO) mice. Female WT displayed mostly mild degenerative changes, with preservation of proteoglycan content. In contrast, severely degenerated IVDs from male WT mice exhibited chondrocyte-like cells distributed within a fibrocartilaginous matrix and reduced Alcian blue staining in the NP. (**b**,**c**) Quantification of histological scores. A *p*-value < 0.05 was considered statistically significant; Female IVDs: WT n = 12, KO n = 16; Male IVDs: WT n = 10, KO n = 12.

We previously demonstrated that *Phlpp1* depletion protected against spontaneous IDD in aged 20-month-old mice, which corresponds to 50–60-year-old humans [42]. In the present study, this same mouse cohort was analyzed separately by sex to evaluate female and male mice independently. Histological analysis showed that 80% of lumbar IVDs from female and male WT mice exhibited degenerative changes (Table 4). Female WT mice developed primarily mild IDD (IVD score 2–6), characterized by NP degeneration, including fibrotic tissue accumulation, matrix disorganization, and cell loss (Figure 2). Notably, severe IDD (IVD score 10–14) was observed exclusively in male WT mice and was absent in female mice, indicating a significant sex-specific difference in IDD severity. This advanced degeneration was characterized by chondrocyte-like cells embedded within a fibrocartilaginous matrix and reduced glycosaminoglycan content in the NP. Conversely, 75% of IVDs from both female and male KO mice remained healthy (score 0–1), which was characterized by a central notochordal NP cell band embedded in proteoglycans of the NP and surrounded by a well-organized collagenous annulus fibrosus, while the remaining 30% displayed only mild degenerative changes. Together, these findings demonstrate that male WT mice display more severe degeneration than female mice, which was attenuated by *Phlpp1* depletion.

### 3.2. Male Phlpp1 Knockout Decreased IL1B and IL6 Expression in Age-Related Spontaneous IDD

**Figure 3:**
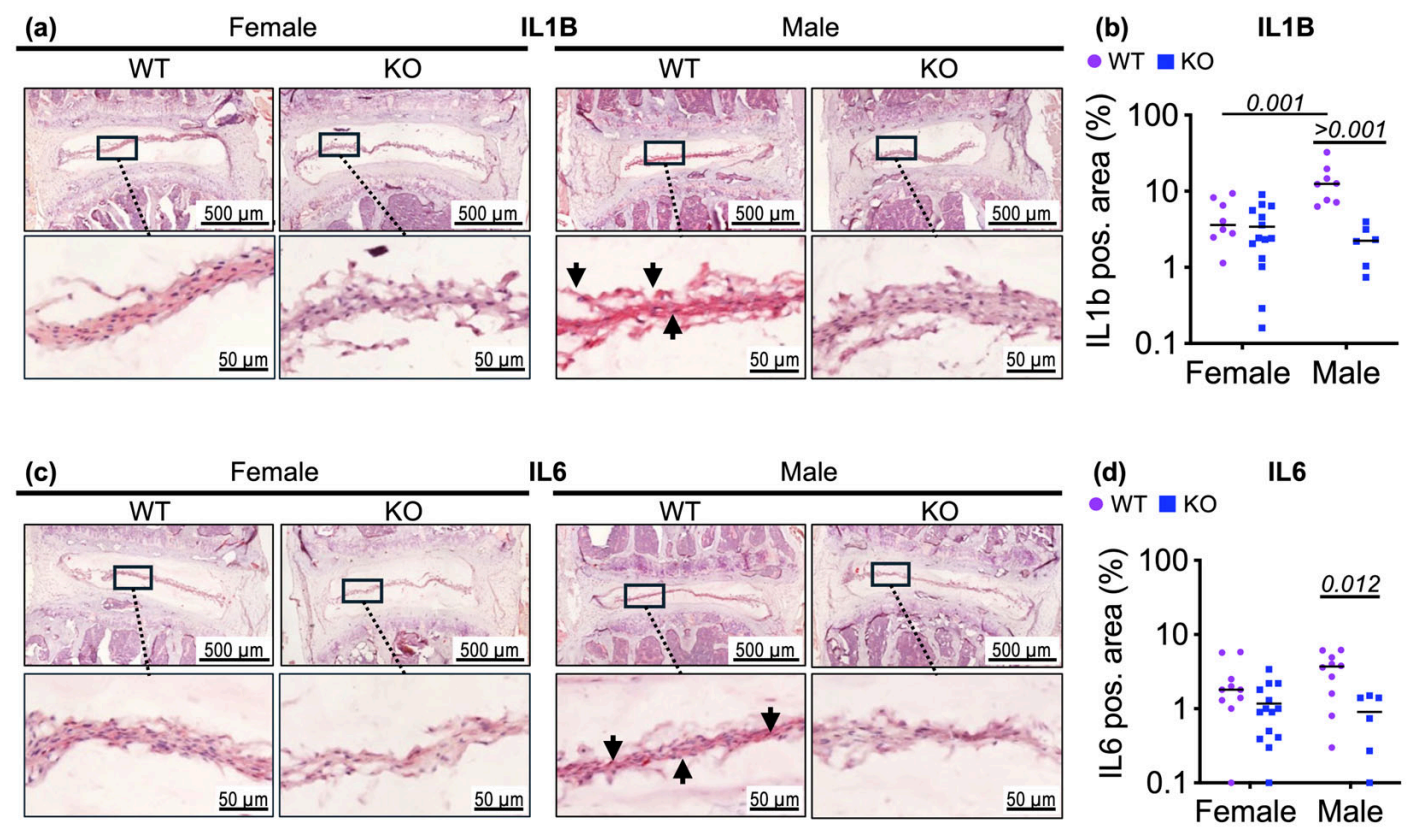
PHLPP1 depletion mitigated inflammation in aging mice. The Proinflammatory cytokines (**a**) IL1B and (**b**) IL6 were down regulated in the NPs of *Phlpp1* deficient mice, which was signidicant in male mice. Representative immunostaining images of (**a**) IL1B and (**c**) IL6 in 20-month-old mouse IVDs (red = positive cells; purple = nuclei); (**b,d**) Graphs represent the quantification of (**b**) IL1B and (**d**) IL6. A p-value < *0.05* was considered statistically significant. IL1B: Female IVDs: WT: n = 9, KO: n = 18; Male IVDs: WT: n = 9, KO: n = 8; IL6: Female IVDs: WT: n = 11, KO: n = 17; Male IVDs: WT: *n* = 11, KO: n = 8. Boxes and lines mark region of interest in the respective images. Arrows denote (**a**) IL1B and (**c**) IL6 positive cells.

We next assessed if the improved histopathological scoring in the NP of KO mice was concomitant with reduced inflammation. Immunohistochemical analysis of IL1B and IL6 revealed that both cytokines were decreased in the NP of male KO mice compared to male WT controls. Noteworthy, for IL1B these changes were partly driven by increased IL1B expression in male compared to female WT mice (Figure 3). Together, our mouse data suggest that *Phlpp1* depletion delays spontaneous IDD in male mice by inhibiting pro-inflammatory signaling.

### 3.3. PHLPP1 Knockdown Altered the Global Transcriptomic Landscape of Human NP Cells

**Figure 4:**
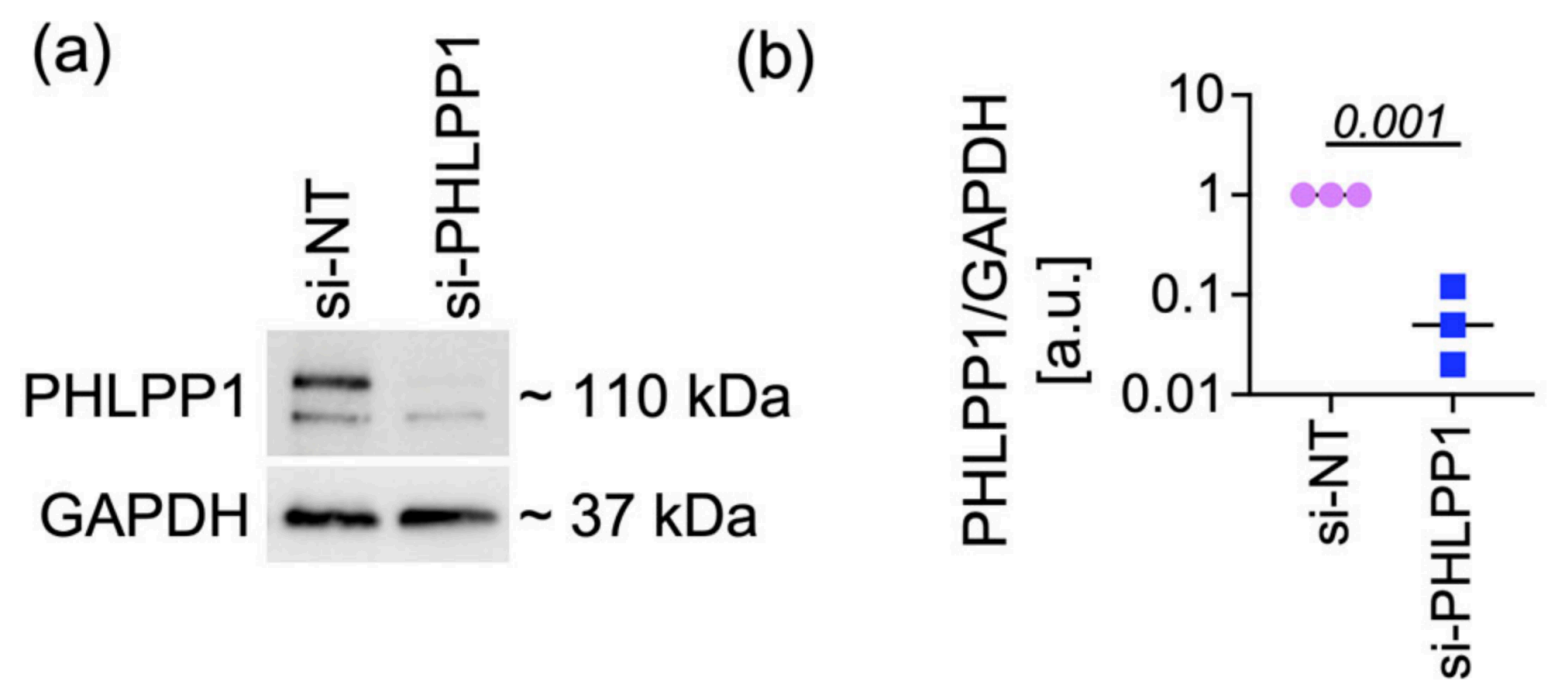
Immunoblotting confirmed *PHLPP1* knockdown. (**a**) Western blot analysis of PHLPP1 protein expression in si-NT and si-PHLPP1 human NP cells. (**b**) Graph represents the quantification of three independent experiments. Values are expressed as mean relative units of PHLPP1 divided by GAPDH and normalized to si-NT.

**Figure 5:**
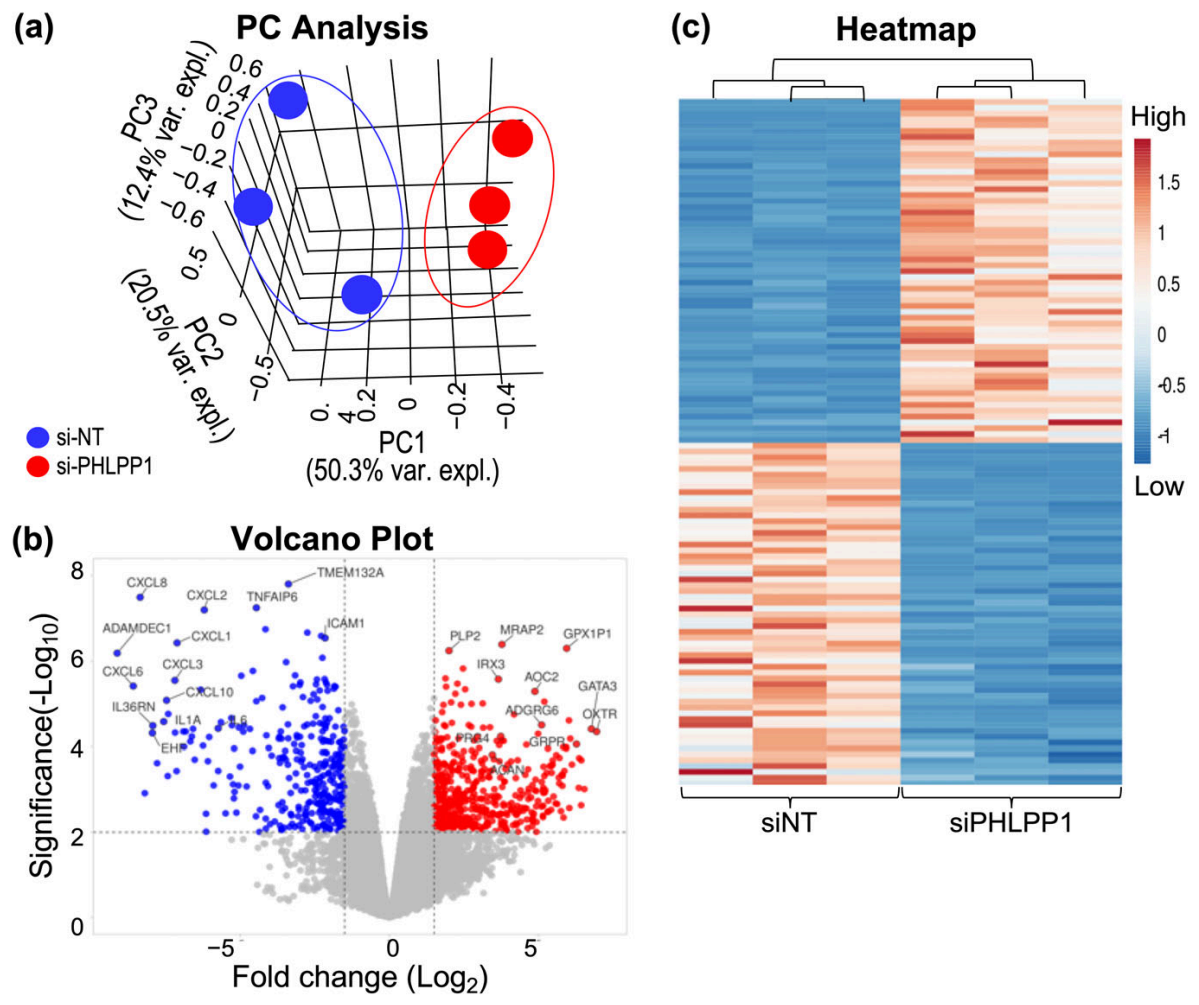
Transcriptomic profiling of human nucleus pulposus cells following *PHLPP1* knockdown. (**a**) Principal component analysis (PCA) showing distinct transcriptomic separation between si-NT and si-PHLPP1 samples. RNA-seq analysis was performed using 3 biological samples per treatment group derived from 4 independent human donors (Table 2); (**b**) Volcano plot of differentially expressed genes following *PHLPP1* knockdown. Red and blue dots indicate significantly upregulated and downregulated genes, respectively; 561 genes were upregulated and 1062 genes were downregulated in si-PHLPP1 samples compared with si-NT controls; (**c**) Heatmap of differentially expressed genes showing hierarchical clustering and transcriptional reprogramming in si-NT versus si-PHLPP1 samples. Red indicates higher expression and blue indicates lower expression.

Although our in vivo data indicated a significantly higher incidence of IDD in male than in female WT mice, human studies consistently report a higher prevalence of IDD in middle-aged females compared to males. To assess the relevance of PHLPP1 as a regulator of pro-inflammatory signaling in human NP cells, we used primary NP cells isolated from degenerated human IVDs obtained from both female and male donors (Table 2). After validating successful knockdown of PHLPP1 following siRNA-mediated knockdown of *PHLPP*1 compared to a non-targeting control siRNA (Figure 4), we performed bulk RNA sequencing (Figure 5). PCA revealed clear segregation between si-NT and si-PHLPP1 groups, indicating that *PHLPP1* knockdown significantly altered the global transcriptional profile of NP cells (Figure 5a). Differential gene expression analysis identified 1062 downregulated genes and 561 upregulated genes in si-PHLPP1 cells compared to control cells (as shown in Volcano plot, Figure 5b).

To further highlight the most strongly regulated genes following *PHLPP1* knockdown, the top 30 upregulated and top 30 downregulated genes are summarized in Table 4 and Table 5, respectively. Among the most significantly downregulated genes were several key inflammatory mediators associated with IDD, including *CXCL1*, *CXCL2*, *CXCL3*, *CXCL5*, *CXCL6*, *CXCL8*, *CXCL10*, *CCL5*, *IL1A*, *IL1B,* and *IL6*, as well as the matrix remodeling enzyme *MMP12* (Table 6). Conversely, genes upregulated following *PHLPP1* knockdown included regulators associated with ECM organization and cellular homeostasis, such as *ACAN*, *PRG4*, *LGR5*, and *MAP1LC3C* (Table 5). These findings support the role of *PHLPP1* in promoting inflammatory signaling and catabolic pathways in NP cells. Hierarchical clustering analysis further demonstrated distinct gene expression patterns between the two groups (Figure 5c), confirming that *PHLPP1* knockdown induced widespread transcriptional reprogramming. Notably, several inflammatory mediators including *CXCL1*, *CXCL2*, *CXCL3*, *CXCL5*, *CXCL6*, *CXCL8*, *CXCL10*, *CCL5*, *IL1A*, and *IL1B*, and *IL6* were among the most significantly downregulated genes following *PHLPP1* knockdown, further supporting our hypothesis that *PHLPP1* regulates inflammatory signaling in NP cells.

### 3.4. PHLPP1 Regulated Inflammatory and Chemokine Signaling Pathways in NP Cells

**Figure 6:**
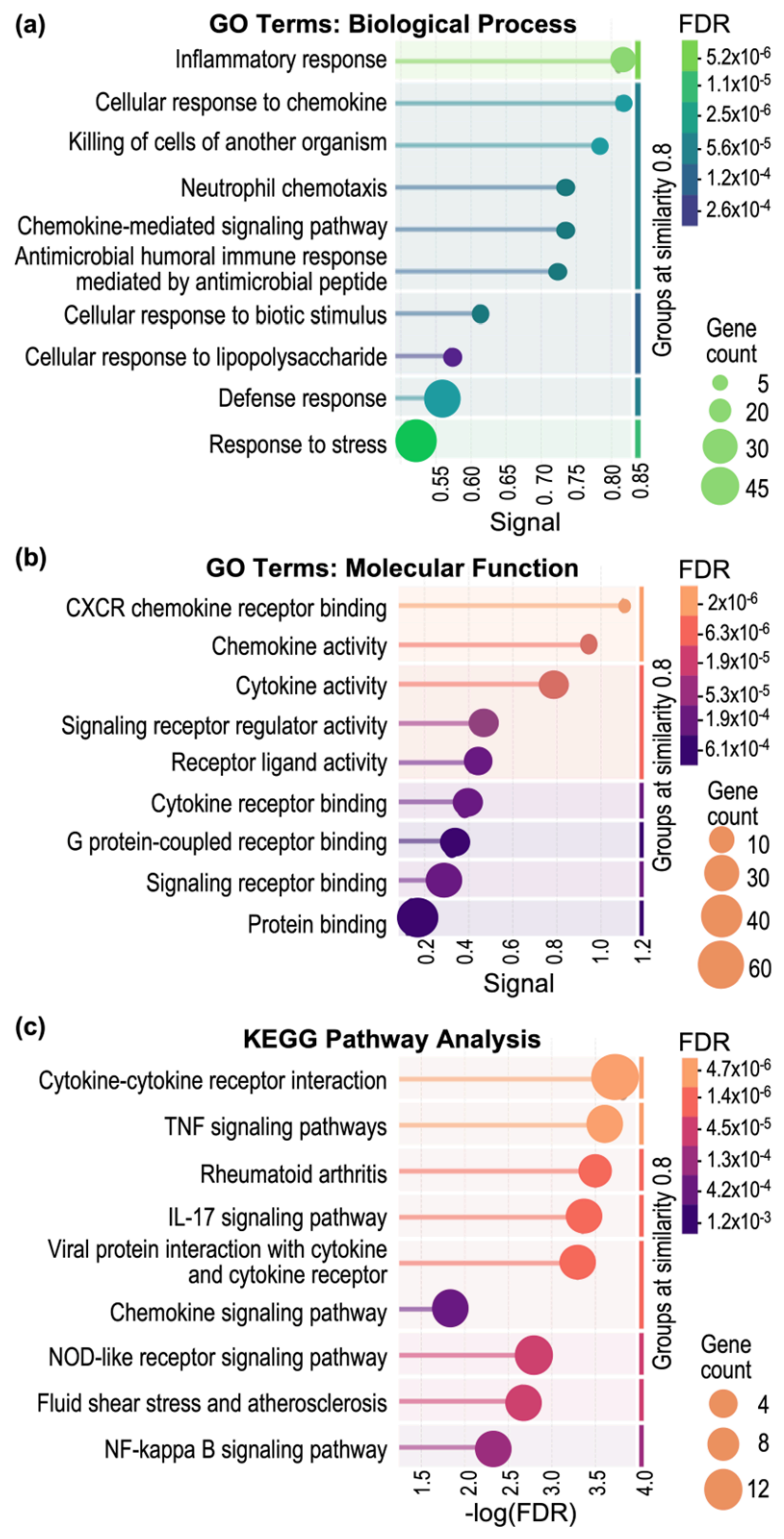
Enrichment analysis of *PHLPP1*-regulated genes. (**a**,**b**) Gene ontology (GO) enrichment of (**a**) molecular function (MF) and (**b**) biological process (BP). (**a**) MF analysis revealed enrichment of genes associated with chemokine receptor binding, chemokine activity, cytokine activity, receptor ligand activity, cytokine receptor binding, and G protein–coupled receptor binding. (**b**) BP analysis demonstrated enrichment of pathways involved in inflammatory response, cellular response to chemokines, neutrophil chemotaxis, chemokine-mediated signaling, antimicrobial immune response, defense response, and response to stress. (**c**) KEGG pathway enrichment analysis identified significant enrichment of cytokine–cytokine receptor interaction, TNF signaling, IL-17 signaling, chemokine signaling, NOD-like receptor signaling, NFKB signaling, and rheumatoid arthritis–related pathways. Dot size represents gene count, and color indicates false discovery rate (FDR) significance.

To determine the biological functions associated with *PHLPP1*-regulated genes, we performed GO enrichment analysis. GO biological process (BP) analysis revealed that the most significantly enriched pathways among downregulated genes were related to inflammatory response, cellular response to chemokines, neutrophil chemotaxis, chemokine-mediated signaling, and defense response (Figure 6a). Consistent with these findings, GO molecular function (MF) analysis identified enrichment of chemokine receptor binding, cytokine activity, receptor ligand activity, and cytokine receptor binding (Figure 6b), indicating that *PHLPP1* plays an important role in regulating cytokine and chemokine signaling networks in NP cells.

### 3.5. KEGG Pathway Analysis Identified Inflammatory Signaling Pathways Downstream of PHLPP1

**Figure 7:**
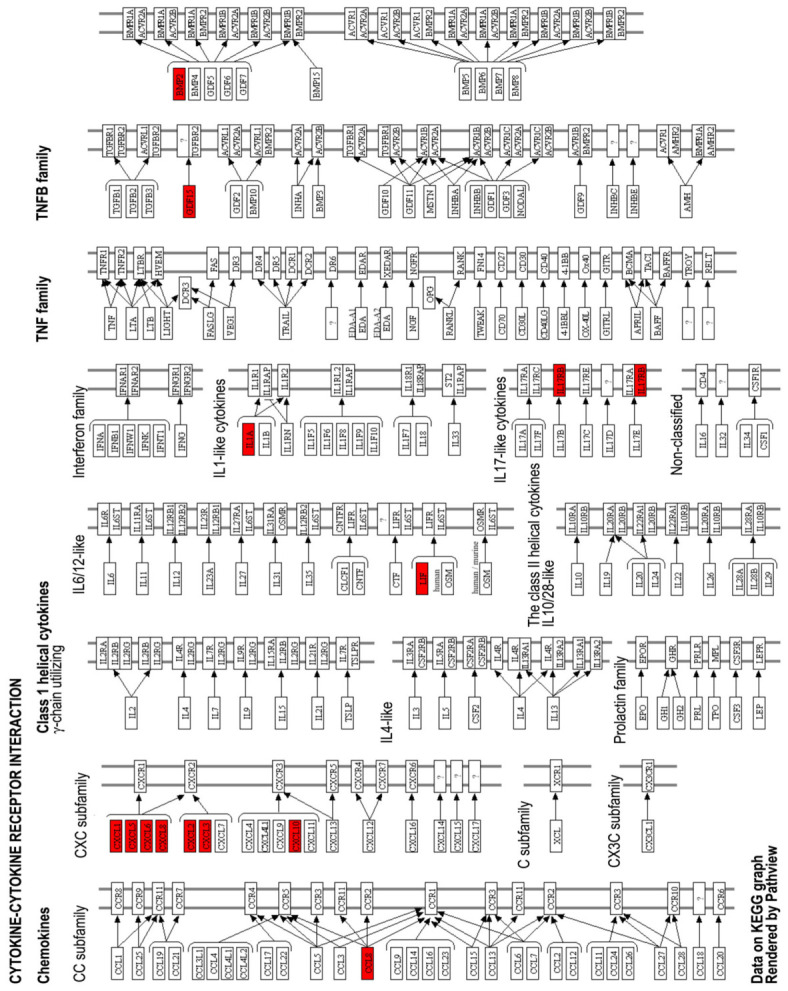
KEGG pathway enrichment analysis identifies inflammatory signaling pathways regulated by *PHLPP1*. Mapping of differentially expressed genes onto the KEGG cytokine–cytokine receptor interaction pathway (hsa04060). Genes significantly downregulated following *PHLPP1* knockdown are highlighted in red. These include multiple chemokines (CXCL1, CXCL2, CXCL3, CXCL5, CXCL6, CXCL8), cytokines (IL1A), and inflammatory signaling molecules, indicating suppression of inflammatory signaling networks in NP cells following PHLPP1 depletion.

KEGG pathway enrichment analysis further revealed that *PHLPP1*-regulated genes are strongly associated with inflammatory signaling pathways (Figure 6c). The most significantly enriched pathways included cytokine–cytokine receptor interaction, TNF signaling pathway, IL17 signaling pathway, chemokine signaling pathway, NOD-like receptor signaling, and NFKB signaling. Mapping the DEGs onto the KEGG cytokine–cytokine receptor interaction pathway demonstrated that multiple chemokines and cytokines, including *CXCL1*, *CXCL2*, *C-XCL3*, *CXCL5*, *CXCL6*, *CXCL8*, *IL1A*, *IL1B*, and *IL17RB*, were significantly suppressed following *PHLPP1* knockdown (Figure 7). These findings suggest that *PHLPP1* regulates a broad inflammatory transcriptional program in NP cells.

### 3.6. Protein Interaction Network Analysis Identifies Coordinated Inflammatory Modules Regulated by PHLPP1

**Figure 8:**
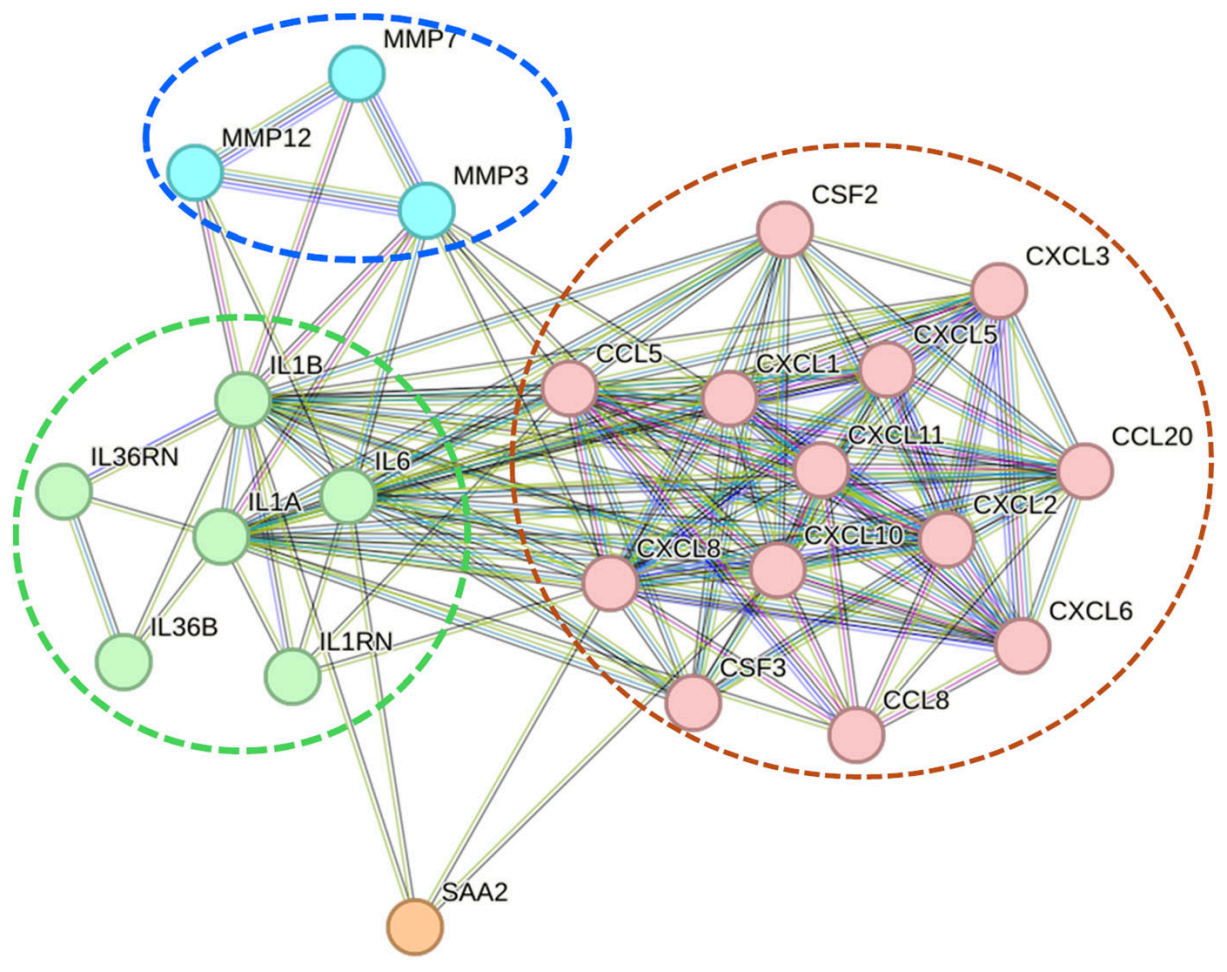
Protein–protein interaction (PPI) network of genes downregulated following *PHLPP1* knockdown. The network was generated using STRING and visualized in Cytoscape (https://cytoscape.org/). Three major functional modules were identified: (1) a chemokine signaling cluster (orange) containing CXCL and CCL family members involved in immune cell recruitment and inflammatory signaling; (2) a cytokine amplification axis (green) including IL1A, IL1B, IL6, IL36B, IL36RN, and IL1RN, representing a central inflammatory signaling hub; and (3) a matrix remodeling module (blue) composed of MMP3, MMP7, and MMP12, key mediators of extracellular matrix degradation in IDD. Together, these interconnected clusters demonstrate that PHLPP1 regulates coordinated inflammatory and matrix remodeling pathways implicated in IDD.

To further understand the functional relationships among PHLPP1-regulated genes, we constructed a protein–protein interaction network using downregulated genes. Network analysis revealed three major interconnected gene clusters (Figure 8). The first cluster consisted of chemokines, including CXCL1, CXCL2, CXCL3, CXCL5, CXCL6, CXCL8, CXCL10, CXCL11, CCL5, and CCL20, suggesting that PHLPP1 regulates immune cell recruitment and inflammatory signaling in NP cells. The second cluster represented a cytokine amplification axis, centered around IL1A, IL1B, IL6, and IL36 family members, indicating a role for PHLPP1 in modulating cytokine signaling. The third cluster consisted of matrix remodeling genes, including MMP3, MMP7, and MMP12, which are well-known mediators of extracellular matrix degradation during IDD. Together, these findings indicate that PHLPP1 regulates a coordinated inflammatory signaling network linking cytokine signaling, chemokine-mediated immune recruitment, and matrix remodeling pathways in NP cells.

### 3.7. PHLPP1 Knockdown Reduced Inflammation and Increased ECM Synthesis in Degenerated Human NP Cells

**Figure 9:**
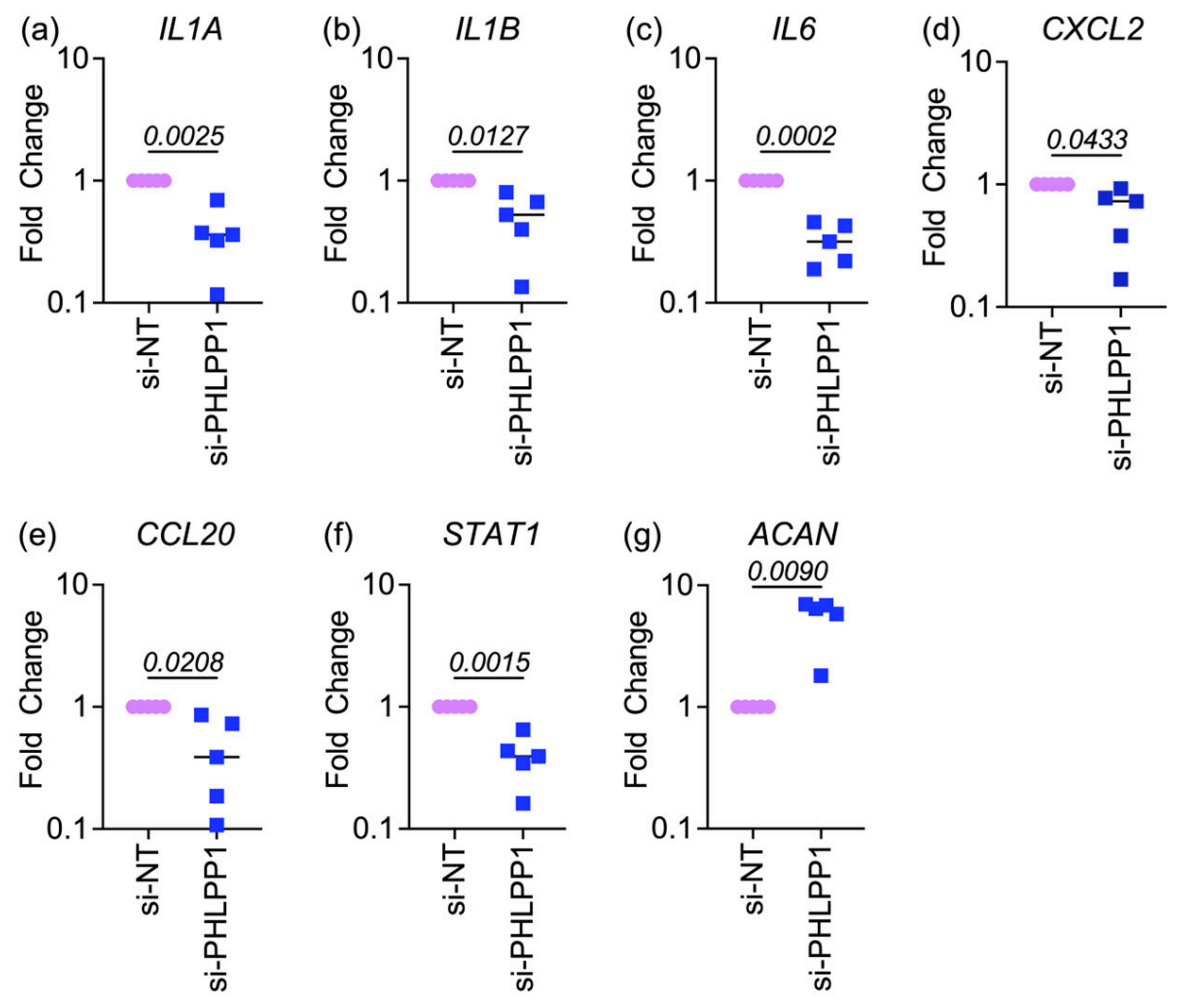
qPCR validated bulk RNA-sequencing findings and confirmed the protective effect of PHLPP1 against inflammation and matrix degradation. Gene expression of (**a**) *IL1A* (**b**) *IL1B*, (**c**) IL6 (**d**) *CXCL2*, (**e**) *CCL20* and (**f**) *STAT1* was significantly decreased after *PHLPP1* knockdown, while (**g**) *ACAN* expression was increased. Data are presented on a logarithmic scale. A *p*-value < 0.05 was considered statistically significant. N = 5/group.

To validate the bulk RNA-sequencing findings, RT-qPCR was performed on selected genes identified as differentially expressed in si-PHLPP1 NP cells. Consistent with the RNA-seq data, RT-qPCR analysis demonstrated *PHLPP1* deficiency promoted a significant reduction in the expression of the pro-inflammatory cytokines *IL1A*, *IL1B*, and *IL6* (Figure 9a–c), the chemokines *CXCL2* and *CCL20* (Figure 9c,d) and the pro-inflammatory mediator *STAT1* (Figure 9f); while the expression of the ECM proteoglycan *ACAN* (Figure 9e) was significantly increased.

### 3.8. PHLPP1 Knockdown Protected Against IL1B-Induced Pro-Inflammatory Responses in Degenerated Human NP Cells

**Figure 10:**
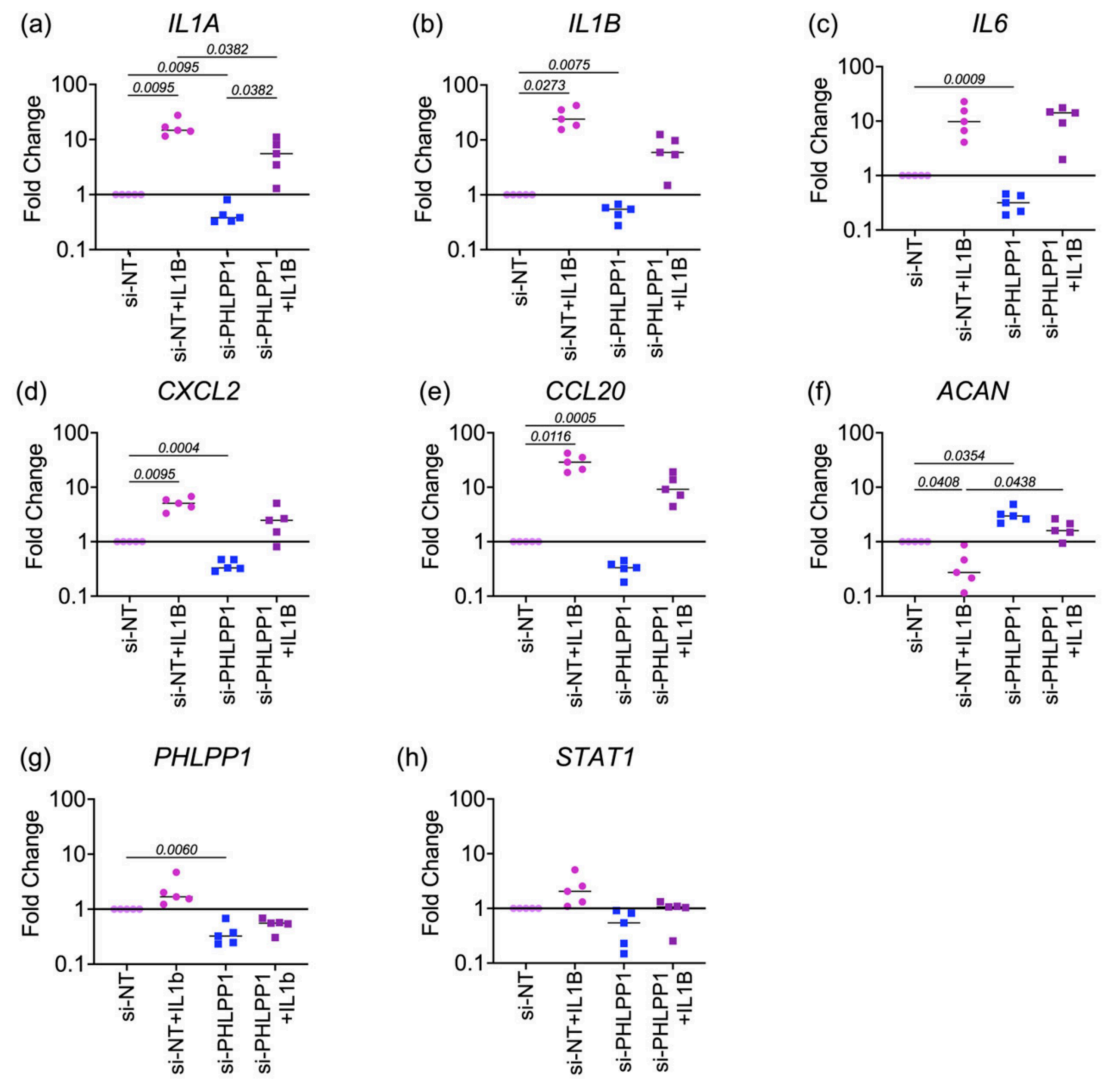
*PHLPP1* silencing attenuated IL1B-induced pro-inflammatory responses in NP cells. Relative to untreated control cells (si-NT), IL1B treatment (si-NT + IL1B) significantly increased expression of the inflammatory markers (**a**) *IL1A*, (**b**) *IL1B*, (**c**) IL6, (**d**) *CXCL2*, and (**e**) *CCL20*, while significantly decreasing expression of (**f**) ACAN. In contrast, IL1B exposure did not significantly alter expression of (**g**) *PHLPP1* or (**h**) *STAT1*. *PHLPP1* knockdown (si-PHLPP1) mitigated the pro-inflammatory and anti-anabolic effects induced by IL1B. Data are presented on a logarithmic scale. Statistical significance was defined as *p <* 0.05. N = 5/group.

**Figure 11:**
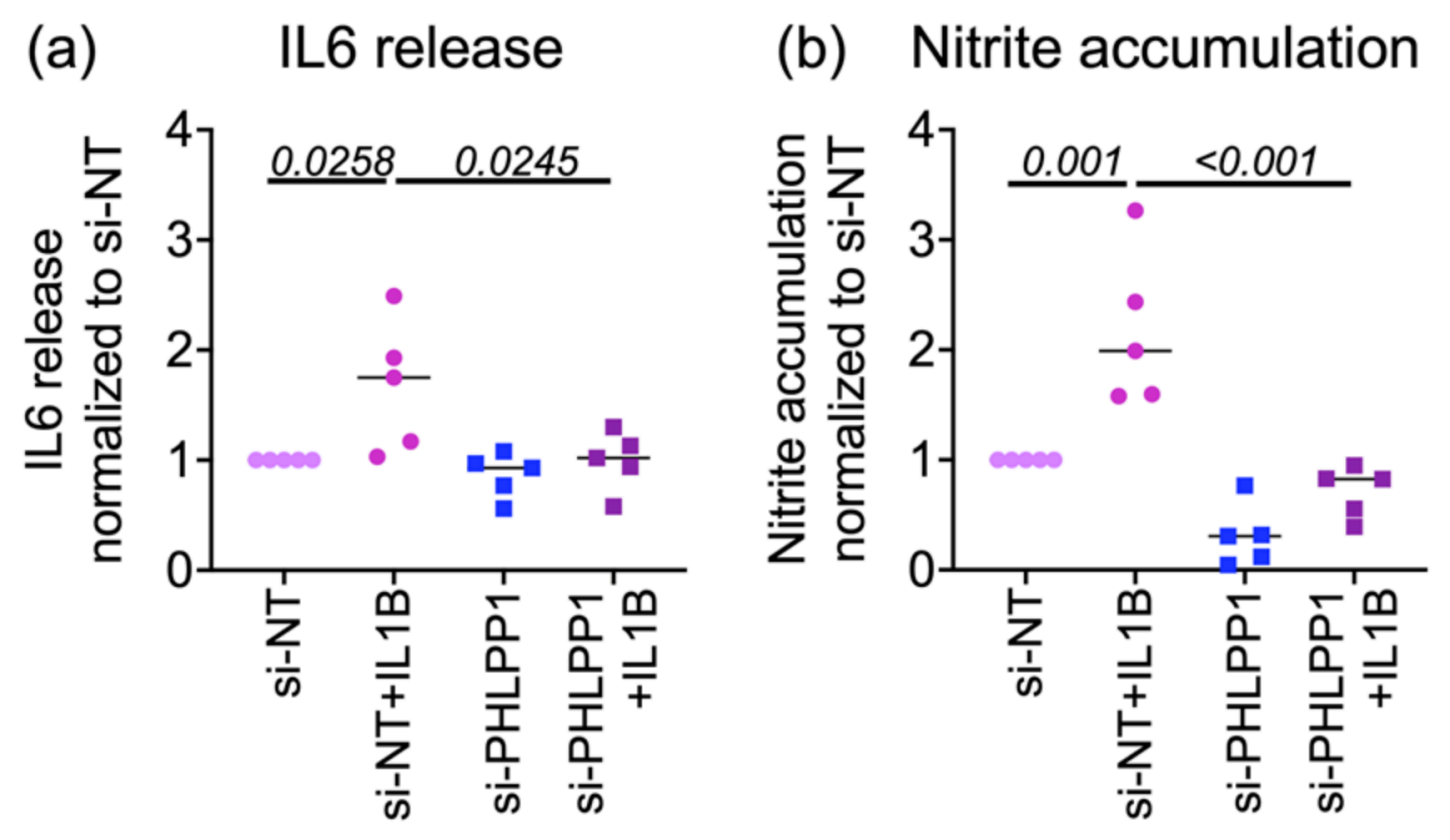
PHLPP1 knockdown attenuated IL6 secretion and nitrite accumulation following IL1B stimulation. IL1B treatment (si-NT + IL1B) significantly increased IL6 secretion and nitrite accumulation compared with untreated control cells (si-NT). Quantification of conditioned media demonstrated that si-PHLPP1 significantly reduced (**a**) IL6 release and (**b**) nitrite accumulation of nitrite in IL1B-stimulated NP cells. Statistical significance was defined as *p* < 0.05. N = 5/group.

To determine whether PHLPP1 regulates inflammatory signaling or is itself regulated by inflammatory stimuli, we stimulated degenerated human NP cells with IL1B, an established model to induce pro-inflammatory responses of NP cells in vitro. As expected, 6 h IL1B exposure significantly increased the expression of the cytokines *IL1A*, *IL1B* and *IL6*, the chemokines *CXCL2* and *CCL20*, while decreasing the expression of *ACAN* in degenerated human si-NT NP cells (Figure 10). In contrast, si-PHLPP1 mitigated the pro-inflammatory and anti-anabolic effects induced by IL1B, suggesting that PHLPP1 functions as a regulator of inflammatory signaling rather than a downstream target of inflammatory stimulation. Consistent with these transcriptional changes, IL6 ELISA and nitrite measurements (Griess assay) showed a comparable pattern, indicating that gene expression changes are reflected at the protein level and supporting a coordinated suppression of inflammatory signaling upon *PHLPP1* silencing (Figure 11).

### 3.9. PHLPP1 Silencing Suppressed STAT1 Activation During Inflammatory Stimulation

**Figure 12:**
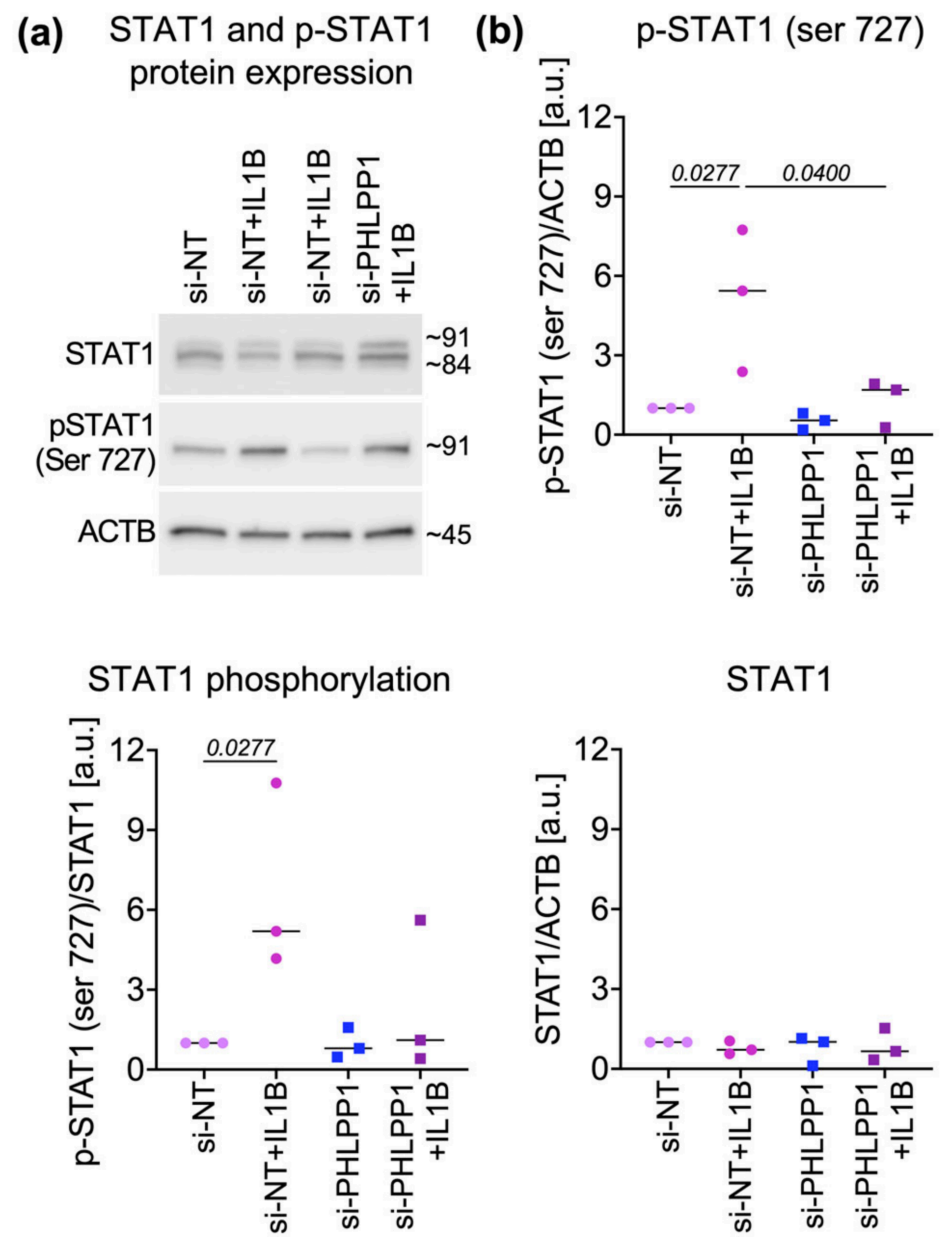
Silencing of PHLPP1 led to a decrease in IL1B-induced STAT1 phosphorylation. IL1B treatment (si-NT + IL1B) significantly increased STAT1 phosphorylation compared with untreated control cells (si-NT). PHLPP1 knockdown (si- PHLPP1) significantly decreased IL1B-induced (si-PHLPP1 +IL1B) STAT1 phosphorylation. (**a**) Western blot analysis of si-PHLPP1 or si-NT degenerated human NP cells with or without 30 min IL1B treatment; (**b**) Graphs represent the quantification of p-STAT1 (ser 727), total STAT1, and relative STAT1 phosphorylation. Values are expressed as the mean of the ratio of STAT1 to ACTB (beta-actin), pSTAT1 (ser727) to ACTB, and p-STAT1 (ser727) to STAT1 and are normalized to si-NT. Statistical significance was defined as *p* < 0.05. N = 3/group.

To further investigate the mechanism underlying the anti-inflammatory effects of *PHLPP1* silencing, we examined STAT1 activation following IL1B stimulation. IL1B exposure for 30 min significantly increased STAT1 phosphorylation in si-NT NP cells relative to untreated si-NT controls. In contrast, *PHLPP1* silencing prevented the IL1B-induced increase in STAT1 phosphorylation, with phospho-STAT1 levels in si-PHLPP1 + IL1B cells remaining comparable to baseline controls. These findings identify STAT1 signaling as a downstream pathway regulated by PHLPP1 during inflammatory stimulation of degenerated human NP cells (Figure 12). Collectively, these findings demonstrate that PHLPP1 promotes IL1B-induced inflammatory signaling in degenerated human NP cells and that its silencing attenuates both downstream inflammatory gene expression and STAT1 activation.

## 4. Discussion

Pro-inflammatory cytokines, including IL1A, IL1B and IL6, are known to promote catabolic activity and contribute to progressive ECM degradation within the IVD [16,43,44]. We previously established that PHLPP1 is highly expressed in degenerated human IVDs and that *Phlpp1* knockout protected against severe IDD in aged mice [22]. Our present study demonstrated that *Phlpp1* deficiency reduced the expression of IL1B and IL6 in the NP of aged male mice. Using degenerated human NP cells for unbiased transcriptomic analysis, we verified *PHLPP1* as an upstream regulator of inflammatory signaling networks. Transcriptional and translational validation confirmed that its deficiency inhibited inflammatory signaling and catabolic pathways in NP cells.

Current evidence for PHLPP1-mediated regulation of inflammation in musculoskeletal tissues remains limited and tissue-dependent [23,25,26]. In cartilage, PHLPP1 expression is induced by pro-inflammatory stimuli and contributes to catabolic responses associated with osteoarthritis [25], whereas in bone it modulates inflammatory signaling in osteoclasts and macrophage-lineage cells, where it influences cytokine production and bone resorption [23,26]. Our findings suggest that PHLPP1 promotes inflammatory signaling in the NP under degenerative conditions. However, IDD is a multifactorial disease, and PHLPP1 upregulation may not universally precede or causally drive degeneration but may instead represent a context-dependent response to inflammatory or degenerative stimuli. The molecular pathways through which PHLPP1 regulates inflammatory signaling within the NP remain poorly defined, necessitating an unbiased approach to comprehensively characterize its downstream effects.

To better define the downstream mechanisms regulated by PHLPP1, we performed RNA sequencing in degenerated human NP cells. *PHLPP1* knockdown profoundly altered the transcriptional landscape, suppressing a broad inflammatory program, including genes encoding inflammatory cytokines, chemokines, and ECM-degrading enzymes (Table 5). While PHLPP1 has been reported to negatively regulate inflammatory signaling through suppression of cytokine expression and dephosphorylation of STAT1 [45]. Our data suggested that *PHLPP1* depletion reduced both *STAT1* gene expression (Figure 9 and Figure 10, Table S1) and STAT1 phosphorylation (Figure 11). This finding suggests a context-dependent role of PHLPP1 in IDD and is consistent with previous transcriptomic studies identifying *STAT1* as a degeneration-associated gene in human NP cells [45]. Thus, reduced STAT1 signaling may contribute to the diminished inflammatory and catabolic gene expression observed following *PHLPP1* knockdown.

Functional enrichment analyses further demonstrated that PHLPP1 regulates key inflammatory pathways associated with IDD, including cytokine-cytokine receptor interaction, TNF signaling, IL17 signaling, and NFKB signaling, all of which are implicated in the pathogenesis of IDD and contribute to immune cell recruitment, matrix catabolism, and NP cell apoptosis [46]. In addition, protein interaction network analysis revealed that PHLPP1 regulated three interconnected inflammatory modules: chemokine signaling, cytokine amplification, and matrix remodeling [46,47,48,49]. Together, these findings suggest that PHLPP1 regulates three major pathological processes that drive IDD progression by contributing to immune cell recruitment, ECM catabolism, and NP cell apoptosis.

RT-qPCR is often regarded as the gold standard for validating findings made in bulk RNA-seq samples [45]. Consistent with our RNA-seq findings, RT-qPCR analysis confirmed that *PHLPP1* knockdown reduced inflammatory cytokine and chemokine expression while increasing *ACAN* expression. Together with our prior findings [22], as well as the present IHC data, these results support the conclusion that the protective effects of *PHLPP1* depletion are mediated, at least in part, through suppression of inflammatory signaling. However, our in vitro experiments were limited to a relatively short stimulation that captures acute responses but does not reflect the chronic nature of IDD. Future studies examining longer-term effects are necessary to determine sustained and adaptive responses to PHLPP1 modulation [45].

Notably, in mice, severe age-associated IDD and inflammation were only observed in IVDs of male WT mice, whereas female mice exhibited only mild degenerative changes. These findings are consistent with previous studies demonstrating sex-dependent differences in both age-associated and injury-induced IDD models, in which male mice generally exhibited greater susceptibility and more severe IDD [50], although these effects may vary depending on model and hormonal context. The sex-dependent effects of *Phlpp1* depletion may similarly be influenced by systemic factors, including hormonal regulation and immune responses. Supporting this possibility, PHLPP1 expression has been shown to be regulated by estrogen in osteoclasts, and its functional effects are altered following hormonal depletion [51], suggesting hormone-dependent modulation of PHLPP1 signaling. However, these mechanisms were not directly investigated in the present study and warrant further investigation.

Several additional limitations should also be considered. IDD is a multifactorial disease influenced not only by molecular regulators such as PHLPP1, but also by aging-associated cellular and molecular alterations, biomechanical loading, genetic susceptibility, lifestyle and environmental factors, and metabolic disorders [52]. Furthermore, although PHLPP1 deficiency attenuated structural degeneration and inflammatory signaling, this study did not assess nerve ingrowth, nociceptive signaling, or pain-related outcomes. Given the established role of aberrant innervation and nociceptor sensitization in discogenic pain, future studies should determine whether the structural and anti-inflammatory benefits of PHLPP1 inhibition translate into reduced pain generation and improved functional outcomes. In addition, the use of global *Phlpp1* knockout mice precludes distinction between NP-specific and systemic effects, although the corresponding results obtained in human cells support a direct role for PHLPP1 within the NP. Future studies using conditional *Phlpp1* knockout models are needed to define cell type-specific mechanisms. Further investigation is also needed to define the mechanisms underlying the observed sex-dependent effects in mice and to evaluate the efficacy of PHLPP1-targeted interventions across diverse patient populations and disease contexts.

Importantly, although severe age-associated degeneration and inflammation were most pronounced in male mice, *PHLPP1* inhibition elicited robust anti-inflammatory and pro-anabolic responses in degenerated human NP cells which contained mainly female donors. While our human donor cohort was not powered to assess sex-specific effects, these findings suggest that *PHLPP1*-regulated pathways are relevant to human IDD beyond the sex-dependent differences observed in mice. The concordance between the protective effects of *Phlpp1* deficiency in mouse IVDs and *PHLPP1* inhibition in human NP cells provides a compelling rationale for continued translational investigation of PHLPP1 as a therapeutic target. Future studies evaluating pharmacological inhibition of PHLPP1, its effects on pain-related outcomes, and its efficacy across sexes will be essential to determine its potential as a disease-modifying therapy for IDD and its associated inflammatory pathology.

## 5. Conclusions

PHLPP1 drives inflammatory and catabolic signaling in IVDs and contributes to age-associated IDD. Genetic depletion in mice and knockdown in degenerated human NP cells suppressed key inflammatory pathways, reduced pro-inflammatory and matrix-degrading mediators, and improved markers of IVD homeostasis. Importantly, these protective effects were conserved across mouse and human models, supporting the clinical relevance of PHLPP1-regulated pathways. Together, our findings identify PHLPP1 as a promising disease-modifying therapeutic target with the potential to slow IDD and improve outcomes for patients with IDD.

## Figures and Tables

**Table 1:** Number of mice/IVDs used for histology and IHC analysis.

	Female WT		Female KO		Male WT		Male KO	
	Mice	IVD	Mice	IVD	Mice	IVD	Mice	IVD
Histology	6	12	8	16	5	10	6	12
IHC—IL1B	4	8	7	14	4	8	3	6
IHC—IL6	5	10	7	14	5	10	3	6

Sample size varies due to limited histological section availability for IHC stains. 2 IVDs/mouse were analyzed (L4/5 and L5/6).

**Table 2:** Patient information.

Patient	Grade	Sex	Age
1	4	F	39
2 *^#^	3/4	M	40
3 *	4	F	53
4 *^#^	3/4	F	59
5 *^#^	4	F	65

* Samples used for RNA-seq; ^#^ Samples used for Immunoblotting.

**Table 3:** Human primer sets used for qRT-PCR analysis.

Gene	Primer Direction	Sequence
*ACTB*	Forward	5′-CGTTTGAGTCAGCAAAGAAGTCAA-3′
	Reverse	5′-CCTTCATGGAGTGGGCCATAG-3′
*IL1A*	Forward	5′-CTCTTCGAGGCACAAGGCA-3′
	Reverse	5′-ATGGCTGCTTCAGACACTTGA-3′
*IL1B*	Forward	5′-AACTGCGCTGCCAGTGCT-3′
	Reverse	5′-CCCATTCTTGAGTGTGGCTA-3′
*CXCL2*	Forward	5 -GGCGAATCAGAAGCAAGCAA-3′
	Reverse	5′-GGATTTGCGCACACAGACAA-3′
*CCL20*	Forward	5′-TGTGGGACTGAAGTTCTTGG-3′
	Reverse	5′-AGCGAGTTGTCATGGTCTG-3′
*ACAN*	Forward	5′-CTCTTCCAGCCTTCCTTCCT-3′
	Reverse	5′-AGCACTGTGTTGGCGTACAG-3′
*STAT1*	Forward	5′-CTGTGAAGTTGAGAGATGTGA-3′
	Reverse	5′-GACCCTCATTCGTTCTGGTG-3′
*PHLPP1*	Forward	5′-TGTGCTGGAGAGGGAAGAGA-3′
	Reverse	5′-AGAGGGCTCCAGTCTCCTGA-3′

**Table 4:** Incidence of IDD in WT and KO mice.

IDD Score: (1–14)		WT				KO			
		Normal (0–1)	Mild (2–6)	Moderate (6–9)	Severe (10–14)	Normal (0–1)	Mild (2–6)	Moderate (6–9)	Severe (10–14)
Female	Mice	1 (16%)	4 (67%)	1 (16%)	- (0%)	6 (75%)	2 (25%)	- (0%)	- (0%)
	IVDs	4 (33%)	7 (58%)	1 (8%)	- (0%)	12 (75%)	8 (25%)	- (0%)	- (0%)
Male	Mice	- (0%)	3 (60%)	- (0%)	2 (40%)	3 (50%)	3 (50%)	- (0%)	- (0%)
	IVDs	2 (20%)	5 (50%)	- (0%)	3 (30%)	9 (75%)	3 (25%)	- (0%)	- (0%)

**Table 5:** Genes that were upregulated following *PHLPP1* knockdown.

SN	Gene	logFC siRNA (Phlpp1/CNT)	FC siRNA (Phlpp1/CNT)	*p*-Value	Adjusted *p*-Value
1	*GPX1P1*	5.94	61.45	5.03 × 10^−7^	7.02 × 10^−4^
2	*CLEC3B*	5.31	39.74	1.11 × 10^−4^	8.28 × 10^−3^
3	*TINAGL1*	5.19	36.58	8.92 × 10^−6^	2.68 × 10^−3^
4	*MSMP*	5.17	35.90	2.93 × 10^−4^	1.34 × 10^−2^
5	*ADGRG6*	5.10	34.33	3.14 × 10^−5^	5.04 × 10^−3^
6	*AOC2*	4.88	29.36	5.14 × 10^−6^	1.93 × 10^−3^
7	*SAMD11*	4.59	24.13	9.00 × 10^−5^	7.53 × 10^−3^
8	*LZTS1*	4.30	19.70	2.06 × 10^−4^	1.13 × 10^−2^
9	*CILP*	4.09	16.99	2.92 × 10^−3^	4.63 × 10^−2^
10	*STEAP4*	3.97	15.62	2.60 × 10^−4^	1.25 × 10^−2^
11	*MAP1LC3C*	3.89	14.80	3.28 × 10^−4^	1.44 × 10^−2^
12	*S100A1*	3.85	14.46	6.49 × 10^−4^	2.11 × 10^−2^
13	*LINC02593*	3.83	14.23	7.38 × 10^−5^	6.87 × 10^−3^
14	*MRAP2*	3.77	13.65	4.07 × 10^−7^	6.25 × 10^−4^
15	*PRG4*	3.73	13.31	5.80 × 10^−5^	6.38 × 10^−3^
16	*DYSF*	3.69	12.87	2.18 × 10^−4^	1.16 × 10^−2^
17	*LGR5*	3.68	12.83	9.23 × 10^−5^	7.57 × 10^−3^
18	*IRX3*	3.65	12.59	2.66 × 10^−6^	1.75 × 10^−3^
19	*SCUBE3*	3.60	12.12	7.38 × 10^−5^	6.87 × 10^−3^
20	*PODXL*	3.57	11.90	2.30 × 10^−3^	4.06 × 10^−2^
21	*FIBCD1*	3.51	11.40	1.53 × 10^−3^	3.29 × 10^−2^
22	*DSC3*	3.50	11.33	1.96 × 10^−4^	1.12 × 10^−2^
23	*ACAN*	3.45	10.94	1.55 × 10^−4^	9.99 × 10^−3^
24	*GFAP*	3.44	10.88	1.43 × 10^−3^	3.19 × 10^−2^
25	*AOC3*	3.37	10.35	3.55 × 10^−5^	5.15 × 10^−3^
26	*EFNB2*	3.32	9.95	5.00 × 10^−4^	1.80 × 10^−2^
27	*HTR7*	3.28	9.74	1.92 × 10^−3^	3.67 × 10^−2^
28	*NGEF*	3.27	9.65	1.45 × 10^−3^	3.22 × 10^−2^
29	*NPTX2*	3.22	9.32	3.33 × 10^−3^	4.89 × 10^−2^
30	*COL21A1*	3.19	9.13	3.29 × 10^−4^	1.44 × 10^−2^

**Table 6:** Genes that are downregulated following PHLPP1 knockdown.

SN	Gene	logFC siRNA (Phlpp1/CNT)	FC siRNA (Phlpp1/CNT)	*p*-Value	Adjusted *p*-Value
1	*ADAMDEC1*	−9.12	0.00	6.53 × 10^−7^	7.71 × 10^−4^
2	*CXCL6*	−8.58	0.00	3.85 × 10^−6^	1.75 × 10^−3^
3	*CXCL8*	−8.35	0.00	3.20 × 10^−8^	2.43 × 10^−4^
4	*ABCA8*	−8.20	0.00	1.22 × 10^−3^	2.90 × 10^−2^
5	*EHF*	−7.94	0.00	4.83 × 10^−5^	5.88 × 10^−3^
6	*IL36RN*	−7.93	0.00	3.25 × 10^−5^	5.04 × 10^−3^
7	*CXCL5*	−7.78	0.00	2.42 × 10^−4^	1.23 × 10^−2^
8	*IL1A*	−7.56	0.01	2.61 × 10^−5^	5.01 × 10^−3^
9	*CXCL10*	−7.47	0.01	8.31 × 10^−6^	2.66 × 10^−3^
10	*CCL5*	−7.43	0.01	4.84 × 10^−4^	1.77 × 10^−2^
11	*KYNU*	−7.41	0.01	1.73 × 10^−5^	4.03 × 10^−3^
12	*CXCL3*	−7.19	0.01	2.83 × 10^−6^	1.75 × 10^−3^
13	*CCL20*	−7.18	0.01	4.74 × 10^−5^	5.86 × 10^−3^
14	*IL36B*	−7.13	0.01	3.71 × 10^−4^	1.53 × 10^−2^
15	*CXCL1*	−7.11	0.01	3.74 × 10^−7^	6.25 × 10^−4^
16	*CSF3*	−6.91	0.01	4.47 × 10^−5^	5.71 × 10^−3^
17	*PNLIPRP3*	−6.88	0.01	1.01 × 10^−4^	7.97 × 10^−3^
18	*MMP12*	−6.83	0.01	4.47 × 10^−5^	5.71 × 10^−3^
19	*IL1RN*	−6.67	0.01	7.62 × 10^−5^	6.88 × 10^−3^
20	*CXCL11*	−6.62	0.01	5.82 × 10^−5^	6.38 × 10^−3^
21	*LTF*	−6.58	0.01	3.88 × 10^−5^	5.29 × 10^−3^
22	*IL24*	−6.52	0.01	2.01 × 10^−4^	1.13 × 10^−2^
23	*APCDD1*	−6.32	0.01	4.69 × 10^−6^	1.90 × 10^−3^
24	*MIR3142HG*	−6.25	0.01	9.51 × 10^−5^	7.73 × 10^−3^
25	*CXCL2*	−6.20	0.01	6.34 × 10^−8^	2.43 × 10^−4^
26	*LINC02392*	−6.08	0.01	2.18 × 10^−4^	1.16 × 10^−2^
27	*IL1B*	−6.04	0.02	6.01 × 10^−5^	6.43 × 10^−3^
28	*CSF2*	−5.89	0.02	8.03 × 10^−4^	2.36 × 10^−2^
29	*IL6*	−5.74	0.02	3.77 × 10^−5^	5.27 × 10^−3^
30	*BST2*	−5.74	0.02	3.69 × 10^−4^	1.53 × 10^−2^

## Data Availability

The RNA-sequencing data generated and analyzed in the current study will be made available upon acceptance in the Gene Expression Omnibus (GEO) database with specific accession number.

## Supplementary Materials

The following supporting information can be downloaded at: https://www.mdpi.com/article/10.3390/cells15151362/s1, Figure S1: *PHLPP1* knockdown significantly downregulates *PHLPP1* expression in human NP cells. Table S1: Additional significantly downregulated genes following *PHLPP1* knockdown.

## Abbreviations

The following abbreviations are used in this manuscript:

ACAN	Aggrecan
ACTB	Beta-actin
ADAMTS	A Disintegrin and Metalloproteinase with Thrombospondin Motifs
AF	Annulus Fibrosus
AKT	Protein kinase B
ANOVA	Analysis of Variance
BDY	Vertebral Body
BP	Biological Process
Bp	Base pairs
CCL5/8/20	C-C Motif Chemokine Ligand 5, 8, or 20
cDNA	Complementary DNA
CO2	Carbon Dioxide
CSF2/3	Colony Stimulating Factor 2 or 3 +1
CXCL	C-X-C Motif Chemokine Ligand (e.g., CXCL1, 2, 3, 5, 6, 8, 10, 11)
DEG	Differentially Expressed Gene
DMEM	Dulbecco’s Modified Eagle Medium
ECM	Extracellular Matrix
EP	Cartilaginous Endplate
FBS	Fetal Bovine Serum
FDR	False Discovery Rate
FPKM	Fragments Per Kilobase of transcript per Million mapped reads
GO	Gene Ontology
GPR68	pH-sensing G protein-coupled orphan receptor
IACUC	Institutional Animal Care and Use Committee
IDD	Intervertebral Disc Degeneration
IHC	Immunohistochemistry
IL1A/IL1B	Interleukin 1 Alpha/Interleukin 1 Beta
IL6	Interleukin 6
IL17/IL17RB	Interleukin 17/Interleukin 17 Receptor B
IL1RN	Interleukin 1 Receptor Antagonist
IL36B/IL36RN	Interleukin 36 Beta/Interleukin 36 Receptor Antagonist
IRB	Institutional Review Board
IVD	Intervertebral Disc
KEGG	Kyoto Encyclopedia of Genes and Genomes
KO	Knockout
L3-L6	Lumbar vertebrae levels 3 through 6
LGR5	Leucine-Rich Repeat Containing G Protein-Coupled Receptor 5
MAP1LC3C	Microtubule Associated Protein 1 Light Chain 3 Gamma
MF	Molecular Function
MMP3/7/12	Matrix Metalloproteinase 3, 7, or 12
mRNA	Messenger RNA
NFKB	Nuclear Factor Kappa B
NIH	National Institutes of Health
NLRP3	NLR family pyrin domain containing 3
NP	Nucleus Pulposus
Nrf2	Nuclear factor erythroid 2-related factor 2
ORS	Orthopaedic Research Society
p38/MAPK	p38 mitogen-activated protein kinase
PBS	Phosphate-buffered saline
PCA	Principal Component Analysis
pen/strep	Penicillin and Streptomycin
PHLPP1	PH Domain Leucine-Rich Repeat Protein Phosphatase 1
PI3K	Phosphoinositide 3-kinase
poly(A)	Polyadenylated
PP2A	Protein Phosphatase 2
PPI	Protein–Protein Interaction
PR/AB	Picrosirius red/alcian blue
PRG4	Proteoglycan 4
PTEN	Phosphatase and Tensin Homolog
RT-qPCR	Reverse Transcription Quantitative Polymerase Chain Reaction
RIN	RNA Integrity Number
RUNX1	Runt-related transcription factor 1
SAA2	Serum Amyloid A2
si-NT	Non-targeting siRNA
si-PHLPP1	PHLPP1-targeting siRNA
siRNA	Small Interfering RNA
SIRT1	Sirtuin 1
STAT1	Signal Transducer and Activator of Transcription 1
STRING	Search Tool for the Retrieval of Interacting Genes/Proteins
SVD	Singular Value Decomposition
TNF/TNFA	Tumor Necrosis Factor/Tumor Necrosis Factor Alpha
VA	Veterans Affairs
WT	Wild type
