# Supplementary material for: PHLPP1 Regulates Inflammatory Signaling in Degenerated Nucleus Pulposus Cells in Mice and Humans
Source: Cells. 2026 Jul 29;15(15):1362. doi: 10.3390/cells15151362 (PMC13465406; doi:10.3390/cells15151362)

Table S1: Additional significantly downregulated genes following *PHLPP1* knockdown

SN	Gene	logFC siRNA (Phlpp1/CNT)	FC siRNA (Phlpp1/CNT)	P. Value	adj.P. Value
1	STAT1	-1.62	0.33	$1.12x10^{-4}$	$8.33x10^{-3}$
2	TMEM144	-1.61	0.33	$2.98x10^{-4}$	$1.35x10^{-2}$
3	ACSS3	-1.60	0.33	$5.21x10^{-4}$	$1.84x10^{-2}$
4	CHDH	-1.60	0.33	$1.31x10^{-3}$	$3.06x10^{-2}$
5	CAND2	-1.60	0.33	$8.32x10^{-4}$	$2.39x10^{-2}$
6	IGF2R	-1.60	0.33	$1.66x10^{-4}$	$1.03x10^{-2}$
7	HELZ2	-1.59	0.33	$9.13x10^{-5}$	$7.55x10^{-3}$
8	DTNA	-1.59	0.33	$1.53x10^{-3}$	$3.29x10^{-2}$
9	SMCR8	-1.12347	0.458989	$1.53x10^{3}$	$3.29x10^{2}$
10	MFF	-0.67859	0.624777	1.55x103	$3.32x10^{2}$

Figure S1: *PHLPP1* knockdown significantly downregulates *PHLPP1* expression in human NP cells

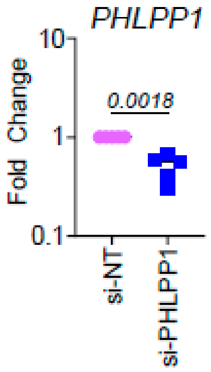

Supplement: Supplementary file 1 [file cells-15-01362-s001.zip › cells-4362661-supplementary.pdf]
